# Emerging Immunogenicity and Genotoxicity Considerations of Adeno-Associated Virus Vector Gene Therapy for Hemophilia

**DOI:** 10.3390/jcm10112471

**Published:** 2021-06-02

**Authors:** Paul E. Monahan, Claude Négrier, Michael Tarantino, Leonard A. Valentino, Federico Mingozzi

**Affiliations:** 1 Hematology, Chapel Hill, NC 27514, USA; pablomonoloco@gmail.com; 2 Department of Hematology, Hospital Louis Pradel, University Claude Bernard Lyon 1, CEDEX, 69677 Bron, France; claude.negrier@chu-lyon.fr; 3 Department of Medicine and Pediatrics, University of Illinois College of Medicine, Peoria, IL 61615, USA; mtarantino@ilbcdi.org; 4 Bleeding and Clotting Disorders Institute, Peoria, IL 61615, USA; 5 National Hemophilia Foundation, New York, NY 10001, USA; lvalentino@hemophilia.org; 6 Department of Pediatric Hematology-Oncology, Rush University Medical Center, Chicago, IL 60026, USA; 7 Spark Therapeutics, Philadelphia, PA 19104, USA

**Keywords:** gene therapy, hemophilia, adeno-associated virus vector, genome integration, liver transduction, immunogenicity, immunologic tolerance, neutralizing antibody, humoral immunity, cellular immunity, innate immunity

## Abstract

Adeno-associated viral (AAV) vector gene therapy has shown promise as a possible cure for hemophilia. However, immune responses directed against AAV vectors remain a hurdle to the broader use of this gene transfer platform. Both innate and adaptive immune responses can affect the safety and efficacy of AAV vector–mediated gene transfer in humans. These immune responses may be triggered by the viral capsid, the vector’s nucleic acid payload, or other vector contaminants or excipients, or by the transgene product encoded by the vector itself. Various preclinical and clinical strategies have been explored to overcome the issues of AAV vector immunogenicity and transgene-related immune responses. Although results of these strategies are encouraging, more efficient approaches are needed to deliver safe, predictable, and durable outcomes for people with hemophilia. In addition to durability, long-term follow-up of gene therapy trial participants will allow us to address potential safety concerns related to vector integration. Herein, we describe the challenges with current methodologies to deliver optimal outcomes for people with hemophilia who choose to undergo AAV vector gene therapy and the potential opportunities to improve on the results.

## 1. Introduction

Adeno-associated viral (AAV) vector gene therapy has shown promise as a possible cure for hemophilia [1]. Early data have demonstrated the potential of this therapy to reduce bleeding and factor VIII (FVIII) or factor IX (FIX) utilization compared with infused blood clotting factor concentrates administered as prophylaxis to prevent bleeding, the standard of care in the developed world [2,3], or when administered on demand as needed to treat acute bleeding episodes, the standard of care in low and low-middle income countries globally. However, immune responses directed against AAV vectors remain a hurdle to the broader use of this gene transfer platform [4].

Evidence has shown that both innate and adaptive immune responses can affect the safety and efficacy of AAV vector–mediated gene transfer in humans, in some cases resulting in acute toxicities. Immune responses may be triggered by various components of the vector, such as the viral capsid, the vector’s nucleic acid payload, or other vector contaminants or excipients, or by the transgene product encoded by the vector itself [4]. Approaches to remediate or prevent immune responses exist, although more effective solutions are needed to address, for example, anti-capsid neutralizing antibodies (NAbs).

The ideal gene therapy delivery system would be safe, not provoke an immune response in humans, and would lead to predictable long-term and durable expression of a therapeutic transgene after a single vector infusion, ameliorating the symptomatology of the underlying disease. For patients with hemophilia (PWH), the goal of gene therapy is to deliver safe, effective, and durable correction of the bleeding diathesis. Importantly, the ideal gene therapy delivery system would be predictable in terms of clinical outcomes, generating consistent results in all PWH A or B.

## 2. Experience with Gene Therapy for Hemophilia to Date

Recent trial results support the potential value of AAV vector–mediated gene therapy for hemophilia [5,6,7,8,9,10,11].

**Figure 1:**
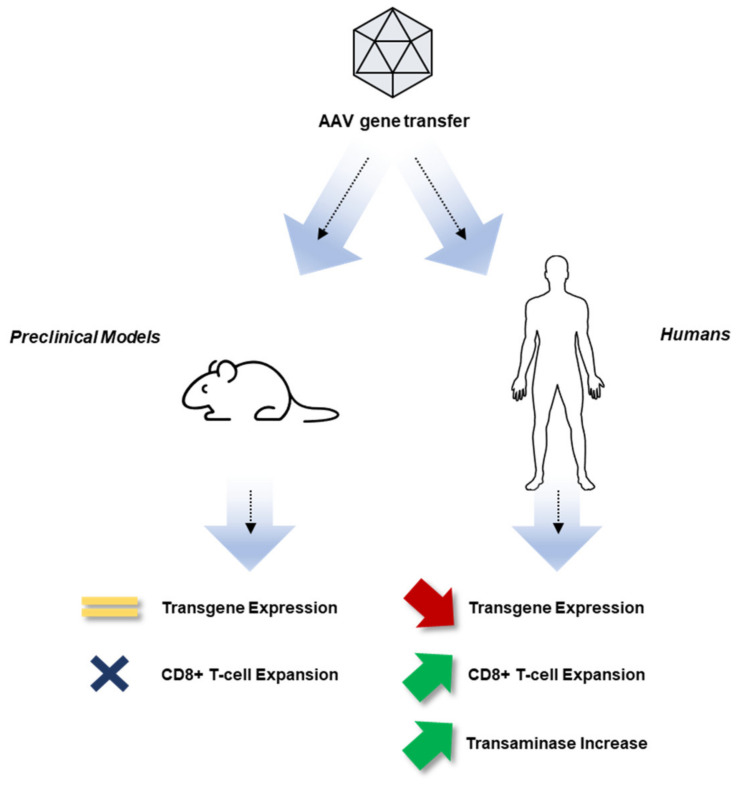
Immune response to AAV gene transfer. In the first gene transfer clinical trial hepatic gene transfer for hemophilia B, an AAV2 vector expressing the human FIX transgene was administered via the hepatic artery. Results in mice and other preclinical animal models showed persistence of transgene expressing and no immune responses following AAV gene transfer. In humans, transgene expression was initially detected but started to decline after 4 to 6 weeks concomitant to an increase in liver enzymes and the detection of T-cell reactivity against the vector capsid. Subsequent studies [16] showed that cytotoxic T-lymphocyte (CTL) expansion detected in the peripheral blood was triggered by the administration of the AAV2 vector in humans and was likely responsible for the clearance of AAV-transduced hepatocytes. As noted, CD8+ T-lymphocyte expansion was not observed in preclinical animal models, in which stable transgene expression had been observed [16]. AAV, adeno-associated virus; FIX, factor IX.

Early attempts to develop an AAV vector-based gene therapy for hemophilia B demonstrated that it was possible to express clotting factors in the human liver at therapeutically relevant levels [10,11,12,13,14,15], although expression was short-lived due to the development of a cytotoxic immune response directed against the vector-transduced hepatocytes [12,16] (Figure 1). In addition, these studies revealed the importance of screening trial participants for preexisting anti-AAV NAbs [12,17]. These studies were the first human trials to focus attention on adaptive immune responses against the AAV capsid and also highlighted the limitations of existing animal models, which had failed to predict these obstacles. In later studies, the use of hepatotropic AAV serotypes [18], combined with transient immunomodulation with oral corticosteroids, resulted in sustained (at least 10 years) expression of human coagulation factor FIX in participants, despite the detection of an immune response directed against the AAV capsid in a subset of participants receiving the vector at the highest dose tested in the study [10,19,20].

This initial success was followed by other trials of AAV vector-based gene therapy in which long-term expression was not consistently achieved [5,21,22], despite the use of immunomodulation with corticosteroids. In a Phase 1/2, open-label, dose-escalation study of BAX 335, an AAV8 vector expressing FIX Padua [14] was administered to people with severe form of hemophilia B. FIX Padua is a naturally occurring missense variant (FIX R338L) with an 8-fold higher FIX-specific activity compared to wild-type (WT) FIX [14,23]. Measurable FIX Padua transgene expression was documented in seven of the eight participants [5], although only one participant achieved sustained therapeutic FIX at approximately 20% of normal, with no bleeding episodes, in the absence of replacement therapy over the 4 years of follow-up. The initial circulating FIX activity was not sustained beyond 5–11 weeks in the other participants in this study, and in some cases, loss of transgene expression was associated with an asymptomatic increase in alanine aminotransferase (ALT) serum concentrations consistent with the clinical observations that accompanied the cytotoxic immune response against vector-transduced hepatocytes in an early trial [12]. Additionally, consistent with previous trials, there was demonstrated to be no development of neutralizing antibodies against FIX and specifically also no antibody development targeting the FIX Padua gain of function variant. However, the use of the same hepatotropic AAV8 serotype (as used in the first successful hemophilia B trial) along with prompt reactive administration of corticosteroids failed to rescue transgene expression in this trial. Additional analyses conducted by these investigators suggested that the loss of transgene expression may have resulted from innate immune responses triggered by the vector genome, more specifically, by the presence of unmethylated cytosine-guanine dinucleotides (CpG) motifs [5], in the sequence of the factor IX expression cassette. Unmethylated CpG motifs are uncommon in mammalian DNA but common in microbial DNA and may induce innate host defenses. As discussed in detail in Section 3 of this review, innate immune sensing is important for instructing adaptive immune responses following microbial infection. While considerable preclinical data in animal models [24,25,26,27,28,29] have explored the possibility that nucleic acid sequence and conformation might contribute to detrimental innate immune signaling in gene therapy (capable of cross-priming adaptive immunity), the outcome of this trial implicated this mechanism in a human recombinant AAV application and prompted re-examination of unexplained lack of efficacy in other trials [30,31]. In the setting of this clinical trial [5] and others in which CpG content was enriched [21,22], the activation of a cytotoxic T-cell response against AAV-transduced hepatocytes was not effectively treated with oral corticosteroid therapy [5].

**Figure 2:**
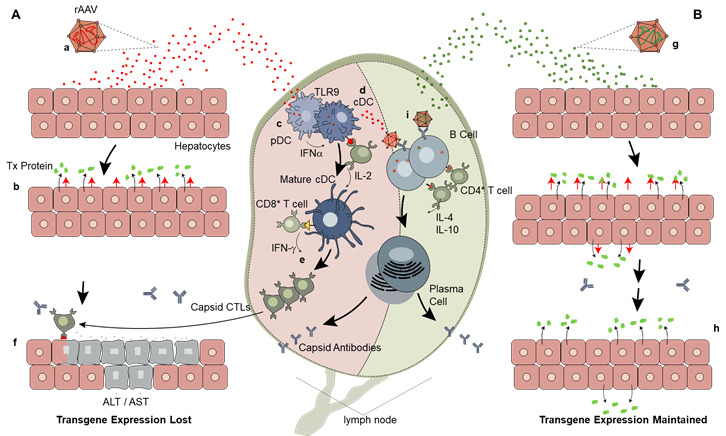
Mechanisms of potential immune responses to AAV vectors [30]. (**A**) Hepatocytes transduced with AAV vectors (a) express the therapeutic protein but also present capsid-derived peptides (yellow triangles) via their MHC class 1 molecules (b). A fraction of the vector dose enters proximal lymph nodes and is taken up by pDCs (c), where vector DNA is processed in the lysosome and promotes the production of proinflammatory cytokines, and by cDCs (d), where vector capsid-derived peptides (red circles) are presented by MHC class 2 molecules, recruiting capsid-specific CD4+ T-cell help. These events lead to licensing and maturation of cDCs and activation of capsid-specific CD8+ CTLs (e) that proliferate, migrate to the liver, and eliminate transduced hepatocytes (f). (**B**) An ideally designed AAV vector with low immunogenicity (g) would similarly transduce hepatocytes but would not activate innate immunity. CTLs would not be formed, transduced hepatocytes would not be eliminated, and cell-surface capsid peptide presentation would wane (h). In both scenarios, AAV vectors activate the humoral arm of the immune response (i), leading to capsid antibodies. Adapted from wright [30]. AAV, adeno-associated virus; ALT, alanine aminotransferase; AST, aspartate aminotransferase; cDC, conventional dendritic cell; CTL, cytotoxic T lymphocyte; IFN, interferon; IL, interleukin; MHC, major histocompatibility complex; PAMP, pathogen-associated molecular pattern; pDC, plasmatoid dendritic cell; TLR9, Toll-like receptor 9; rAAV, recombinant adeno-associated virus; tx, therapeutic.

Recent trials have taken advantage of vector codon optimization (the introduction of synonymous mutations in a nucleotide sequence of a gene expression cassette, resulting in the translation of a conserved amino acid sequence of the transgenic protein) to reduce the content of CpG motifs and thereby reduce the potential for triggering innate immune responses [30]. In hemophilia B gene therapy, this approach has been associated with improving the outcome of gene transfer in terms of managing vector-associated liver inflammation (Figure 2) and achieving sustained transgene expression. George and colleagues [11] showed that an AAV vector expressing the FIX Padua transgene, [14], delivered at relatively low doses (5 × 10^11^ vector genomes per kilogram (vg/kg)) achieved an average FIX activity of 33.7 ± 18.5% (range, 14 to 81%). FIX Padua expression was associated with meaningful reductions in bleeding (mean rate, 9.9 events/year (range, 0 to 48 events/year) before vector administration vs. 0.6 events/year (range, 0 to 4 events/year) after administration in the seven participants in the study previously on prophylaxis). Similarly, sustained transgene expression resulted in a reduction in exogenous factor utilization (mean value, 2908 IU/kg (range, 0 to 8090 IU/kg) before vector administration vs. 49.3 IU/kg (range, 0 to 376 IU/kg) after AAV vector administration). In this study, two of 10 participants experienced an immune-mediated increase in liver enzymes, accompanied by positive IFN-γ ELISPOT directed against AAV-Spark-100 capsid-derived peptides, which was controlled by a short course of oral corticosteroids [11], resulting in sustained correction of the bleeding phenotype. As in the previously described trial, no evidence of immunologic recognition or targeting of the variant FIX Padua protein (or epitopes specific to this mutation) were observed.

Another program in development for hemophilia B used a WT FIX expression construct previously tested in humans [20] and packaged into an AAV5 [9]. Participants enrolled in the Phase 1/2 study received 5 × 10^12^ (*n* = 5) or 2 × 10^13^ vg/kg (*n* = 5) of AAV5-WT FIX gene transfer. Results from the initial one year follow up as well as >4–5 years of follow up have been reported [9,32]. Limited, asymptomatic, transient ALT elevations occurred in 20 and 40% of the participants in the 5 × 10^11^ and 2 × 10^13^ vg/kg cohorts, respectively, and were successfully treated with oral corticosteroids at the time of ALT elevations [9]. Specifically, the periods of liver transaminitis in this trial, which occurred between weeks 4–22 following vector infusion, were characterized by no loss of FIX expression or evidence of circulating T-cells targeting AAV capsid epitopes. Furthermore, the mean long-term sustained levels of factor IX expression in the 5 × 10^12^ vg/kg dose cohort (mean 5.2% FIX activity over 5 years of follow-up) and in the 2 × 10^13^ vg/kg dose cohort (mean 7.4% FIX activity over 4.5 years of follow-up) actually exceeded the mean FIX expression reported at year 1. This program is currently continuing with a modified vector (AMT-061) encoding FIX Padua (Phase 2b trial NCT03489291 [33] and Phase 3 trial NCT03569891). As anticipated, the substitution of the FIX Padua variant has mediated FIX activity that is sustained and that exceeds the activity achieved by the identical doses of the vector expressing WT FIX. From the standpoint of immunology, however, the novel aspect is that subjects who were determined to have pre-existing AAV5 neutralizing antibodies, as measured by the sponsor’s assay methods, were not excluded from participation (see Section 3.1); the sponsor’s preliminary report is provocative in that all subjects with pre-existing AAV5 NAb of low or intermediate titer (up to a titer of >600 using the sponsor’s assay) sustained FIX activity past week 26 (follow-up ongoing) with only one subject with a higher titer NAb failing to show FIX correction [34].

Although several trials of gene therapy for hemophilia A are in progress (Table 1) [42], only one study has published data. In this trial, transgene expression persisted for 3 years in participants receiving an AAV vector that contained a codon-optimized, B domain-deleted factor VIII (FVIII) complementary deoxyribonucleic acid (cDNA; AAV5-hFVIII) [43,44,45]. The multiyear follow-up of AAV5-hFVIII gene therapy found that the treatment significantly reduced annualized rates of bleeding events (ABRs) and resulted in complete cessation of prophylactic FVIII use among participants who received doses of 4 × 10^13^ vg/kg or 6 × 10^13^ vg/kg. Six participants in the 4 × 10^13^–vg/kg dose group had a median FVIII expression of 13 IU/dL 2 years after infusion, while seven participants in the 6 × 10^13^–vg/kg group had a median FVIII expression of 20 IU/dL 3 years after infusion. No inhibitor development, thromboses, or deaths were observed, although changes in liver function tests lasting several weeks were documented; furthermore, the long-term impact of transgene expression has yet to be determined [8,46]. Interestingly, long-term follow-up of participants in this trial highlighted a steady decline in FVIII expression levels [8], which currently remains unexplained. The trial’s sponsor has reported immunogenicity data collected over a follow-up of at least two years (range 104–183 weeks), which confirms that the declining expression does not result from humoral immune response against the FVIII transgene (no FVIII inhibitor development). In addition, while cellular immune responses (as measured by IFN-γ and TNF-α fluorospot with either AAV5 peptide stimulation or FVIII peptide stimulation) were observed intermittently, there was no apparent association between positive responses and changes in liver transaminases or FVIII activity measurements. All subjects had unmeasurable AAV5 NAb at enrollment and developed high titer AAV5 NAb after vector infusion, which persisted at high titer for years and demonstrated cross-neutralization of all other AAV serotypes tested (AAV2, AAV6, AAV8, AAVrh10) [45].

Additional hemophilia A gene therapy trial results have been reported in preliminary form only (Table 1) but when considered in aggregate raise several potential immunologic obstacles and unknowns that will guide future research and development. A consistent reassuring finding across these trials is that FVIII expression has not been associated with FVIII inhibitor development in any trial participant. Nevertheless, sustained expression of FVIII from hepatocytes has been associated with greater interindividual variability in these trials than was experienced in hemophilia B gene therapy. Liver transaminitis and apparent cellular immunity initiating or re-initiating at late time points (more than three to four months following gene transfer) had not been observed in hemophilia B trials but has been reported in several hemophilia A trials [8,35,36,37,38,39,40]. Supportive courses of corticosteroids, whether used in a reactive or prophylactic fashion, have in several trials greatly exceeded the duration used in hemophilia B trials [8,19,35,36,38,39,40], resulting in steroid-associated adverse events and calling into question whether adjunctive or alternative immune modulating agents should be used. A challenge for the field of hemophilia A gene therapy, which provides context for this review, will be to understand to what extent these apparent inflammatory phenomena, as well as the decline in FVIII expression reported in one trial [8], result from immune mechanisms or from mechanisms that are not purely immune in origin (e.g., constraints of synthesis of factor VIII in hepatocytes rather than liver sinusoidal endothelial cells; natural senescence and turnover of hepatocytes) and what mitigating strategies will best balance benefit and risk.

## 3. Overview of the Immune System

**Figure 3:**
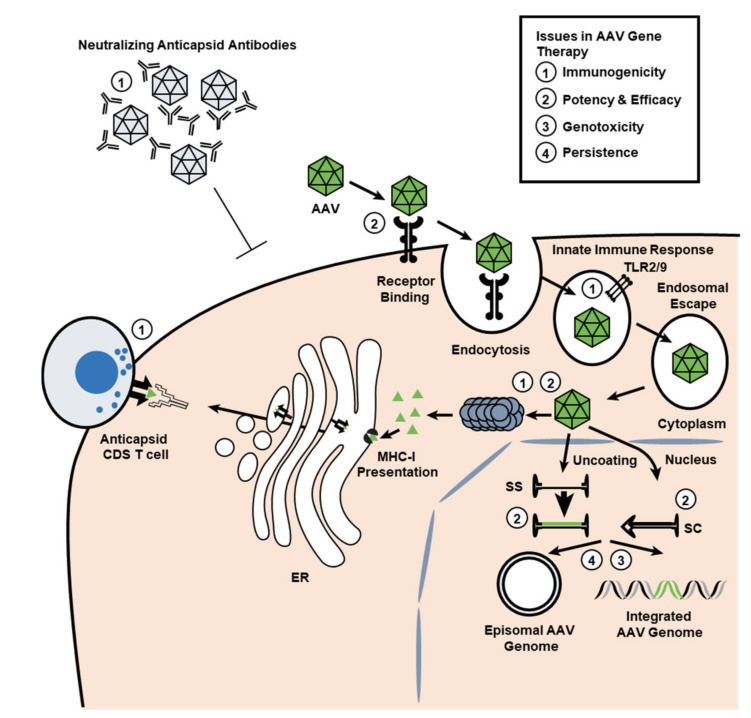
Potential limitations of gene transfer with AAV vectors [47]. Several potential immunologic-related issues to the AAV vector platform are emerging (white box): (1) Vector immunogenicity: the presence of neutralizing antibodies (NAbs) against the AAV capsid can prevent or limit cell transduction, whereas cytotoxic CD8+ T-cell responses can eliminate AAV-transduced cells that present AAV capsid antigens loaded on MHC-I complexes. (2) Potency and efficacy: the efficiency with which AAV vectors infect and transduce into the desired target cells can impact therapeutic doses and efficacy. (3) Genotoxicity: although rare, the integration of the AAV vector DNA into the genome of the infected cell may have genotoxic effects. (4) Persistence: the episomal AAV genome in the nucleus of the infected cells can be lost in conditions of cell proliferation (such as liver growth), which may impact therapeutic efficacy. AAV, adeno-associated virus; ER, endoplasmic reticulum; MHC-I, major histocompatibility complex class I molecule; sc, self-complementary; ss, single strand; TLR, Toll-like receptor.

Immune system response may be innate or adaptive. The adaptive immune response is further divided into humoral (antibody-mediated) immunity, which may manifest as antibodies against the AAV vector or against the product of the expressed transgene, and cellular immunity, which is mediated by T- and B-cell responses (Figure 3).

Variables of the gene delivery approach that may affect the immune response include the virus capsid serotype, the molecular form of the packaged expression cassette content (e.g., single-stranded (ss) or self-complementary (sc) AAV). Other variables include clustering of CpG dinucleotides or other potential pathogen-associated molecular patterns (PAMPs) in the vector capsid or transgene, target tissue, tissue-specificity of the vector’s transcriptional regulatory elements, and dose and purity of the vector [47,48].

Since AAV vectors lack viral coding sequences, the main components that induce an immune response are the viral capsid and the transgene product itself. However, the deoxyribonucleic acid (DNA) component of the AAV vector and the double-stranded ribonucleic acid (dsRNA) produced by the vector may also contribute to activation of the innate response [4,48,49]. Similarly, the AAV capsid itself may interact with the innate immune system [50]. A number of host-dependent factors affect the immune response, including age, human leukocyte antigen (HLA) type, inflammation, and genetic background (e.g., underlying mutations such as large deletions in the *F9* gene, which may influence the formation of anti-FIX inhibitory antibodies) [48,51]. The presence of chronic human immunodeficiency virus (HIV) infection, even if well controlled, may also affect immune responses in gene transfer. Of note, although there has been no transmission of HIV from blood coagulation products in the United States since 1987, before that time, 50% of patients with hemophilia and 90% of patients with severe hemophilia contracted transfusion-associated HIV [52]. Hepatitis also remains highly prevalent in the hemophilia population. Prior to the 1990s, most adults and teens with hemophilia who received transfusions of plasma-derived clotting factor concentrates developed hepatitis infections [53,54]. The impact of hepatitis on the liver microenvironment and the outcome of gene transfer is unclear [55].

### 3.1. Preexisting Immunity to AAV Vectors

Wild-type (WT) AAV is prevalent in the environment, leading to natural AAV infection and consequent seroconversion, commonly during childhood. Humans are the natural host for AAV serotype 2 [56]. Consequently, antibodies that bind and/or neutralize AAV are frequently found in humans, with some variability depending on the serotype analyzed; for example, anti-AAV antibodies to AAV1 and AAV2 are detected in up to 70% of the population, whereas other serotypes have lower seroprevalence [4,57,58]. Humans are the natural host for AAV serotype 2 and in most cases of natural (environmental) exposure to AAV antibodies arise that are likely to cross-neutralize other AAV serotypes [56,59]. Although the response in any given individual may vary, antibodies that arise after exposure to one serotype (e.g., AAV2) may cross-react more strongly with a serotype that is evolutionarily similar (e.g., AAV3) than with a more phylogenetically distinct serotype (e.g., AAV5) [17,18,56,59,60]. Among children, a higher incidence of seropositivity can be found immediately after birth, presumably due to transplacental transfer of maternal antibodies during fetal life [59]. The lowest level of seronegativity can be found in 1 year-olds [59,61]. In children of early school age, the prevalence of NAbs to AAV vectors increases progressively: 43% have NAbs against AAV2, 26% against AAV5, and 23% against AAV8 [59]. Several studies have shown a high prevalence of NAbs to multiple AAV serotypes in the general population as well [62,63]. Confounding the potential understanding of the clinical impact of AAV NAbs is the fact that there is little standardization between laboratories for assays for AAV NAb, as discussed below (see “Adaptive Immunity”). Additionally, large differences in NAb prevalence have been described from different geographic areas, even when identical assay methods are used [62,64,65,66].

**Figure 4:**
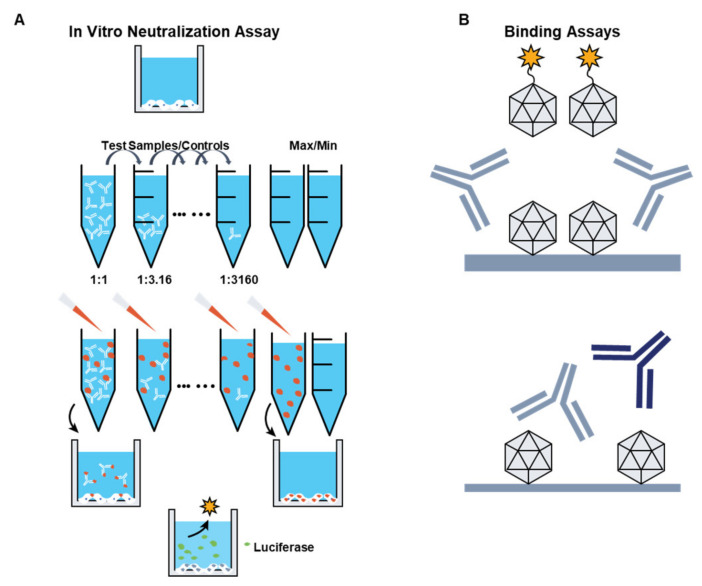
Assays to test for anti-AAV antibodies and neutralizing factors. (**A**) The cell-based transduction inhibition assay measures the ability of plasma samples to reduce the transduction of a cell line by a recombinant adeno-associated virus (rAAV) vector carrying a reporter transgene such as luciferase. In the presence of antibodies, the luciferase-reported fluorescence may be reduced. (**B**) Total anti-AAV antibodies assays on human plasma using a bridging electrochemiluminescence assay or a classic capture assay. AAV capsids are coated to a well, plasma samples are added after blocking, and AAV-specific antibodies are detected using ruthenylated AAV capsids (for electrochemiluminescence assay) or an anti-IgG antibody is added (for the capture assay). max, maximum; min, minimum.

Anti-AAV antibodies can have a profound effect on the efficiency of transduction, particularly in the setting of liver-directed gene transfer [12,67]. The development of sensitive, reliable methods to measure antibody titers is, therefore, critical to screen patients prior to enrollment in gene transfer studies. Several methods have been developed to measure anti-AAV antibodies (Figure 4). Total binding antibodies are typically measured with capture assays, which detect anti-AAV immunoglobulin (Ig) IgG that recognizes the AAV capsid [68]. Neutralizing titers are determined with in vitro assays in which the readout is the vector transduction inhibition activity [69]. Although the determination of total antibody titers relies on assays that are relatively easy to perform, the total amount of anti-AAV antibodies is not always proportional to their neutralizing activity, particularly at low titers [61]. Conversely, anti-AAV neutralization assays provide a sensitive readout of the functional inhibition of transduction, which can be mediated by different immunoglobulins (e.g., IgG, IgM), or other neutralizing factors [57,68]. The detection of anti-AAV neutralizing antibodies in the circulation is a common exclusion criterion in intravenously administered liver-targeted gene therapy studies.

Capsid T-cell responses are less readily detectable than humoral immunity in healthy individuals [11,28,58,70]. One study identified AAV capsid T-cell epitopes in human splenocytes from 44 healthy blood donors and found T-cell responses to the AAV capsid in 16.67% of children under 5 years old and in 73.08% of those ≥5 years old [71]. Another study found AAV-specific T-cells in splenic samples of 62.5 and 57.14% of healthy children and adults, respectively [16]. A study of peripheral blood mononuclear cells (PBMCs) found AAV1 capsid-specific T-cells in 29.1% of healthy adults donors [72], whereas another study found AAV2- or AAV8-specific CD8+ T-cells in the PBMCs of all healthy donors tested [70]. The differences in the frequency of capsid T-cell responses in splenocytes and peripheral blood may reflect the use of different assays and biomarkers used across studies. Multiple studies showed that humoral immune responses do not correlate with interferon gamma (IFN-γ) T-cell responses in healthy donors [16,71,72]. Although several studies have shown that cytotoxic T-cell responses to AAV can be triggered by vector infusion [5,10,11,12,20], resulting in loss of transgene expression, the impact of preexisting T-cell immunity on the outcome of gene transfer remains a matter of debate. Currently, results of baseline evaluation of T-cell reactivity to AAV are not commonly used as exclusion criteria in clinical trials.

### 3.2. Innate Immunity

The innate immune response occurs early and rapidly upon exposure to a pathogen, is predominantly not antigen-specific, and does not result in immunologic memory [4]. However, this response triggers the adaptive immune response through the creation of a pro-inflammatory environment [4,48,49]. Recognition of PAMPS initiates the innate immune response. Pattern recognition receptors (PRRs) on immune cells recognize viral nucleic acids, membrane glycoproteins, and chemical messengers, which leads to activation of nuclear factor kappa B (NF-κB) and interferon (IFN)-regulatory factor transcription factors that induce pro-inflammatory cytokine expression or type I IFNs [4,27,73]. Type I IFNs are thought to play an important role in the stimulation of anti-capsid CD8+ T-cell responses [27]. In nonparenchymal liver cells, such as Kupffer cells and liver sinusoidal endothelial cells (LSECs), the viral capsid activates innate immunity by binding to the Toll-like receptor (TLR) 2 [50], while the double-stranded (ds) DNA vector genome and unmethylated CpG motifs are recognized by TLR9 found in Kupffer cells [24], peripheral plasmacytoid dendritic cells [25,26], and monocyte-derived dendritic cells [74]. TLR9 engagement appears to be involved in capsid-specific CD8+ T-cell activation [25,29,75].

Recent research has demonstrated the role of interleukin (IL)-1β and IL-6 in the innate immune response and the importance of innate immunity in shaping the adaptive immune response [28]. The results of this study also showed that neutralizing IL-1β and IL-6 in PBMCs significantly diminish the frequency of AAV-specific antibody-secreting cells (ASCs) and anti-AAV antibody levels [28].

Although innate recognition of AAV vectors has been described in multiple preclinical models [24,25,26,28,29], the central clinical role of this early arm of the immune system has only recently been appreciated following the report of a hemophilia B trial suggesting the role of TLR9 activation as a trigger for anti-capsid cytotoxic T-cell responses [5]. In this trial, an AAV vector containing a genome with a high number of CpG repeats failed to achieve long-term transgene expression in all subjects but one; this individual had a functional polymorphism in the gene for the IL-6 receptor, which would be expected to disrupt normal pro-inflammatory signaling. Furthermore, an asymptomatic elevation in IL-6 was observed during the hours following AAV vector infusion in one subject from the highest-dose cohort, who did proceed to express transgenic FIX initially but then lost expression of this coagulation factor [5].

A comprehensive overview of vector CpG content of FIX transgene sequences employed in hemophilia B gene therapy trials has recently been published [30]; this analysis suggested a direct relationship between the potential for innate immune activation and clinical outcome (Table 2).

The ability to track the progression of immune events in clinical trials has been limited by the desire to avoid invasive procedures in hemophilia patients, with the result that phenomena that are happening within the liver and at the tissue level may not be detected easily in assays performed on peripheral blood samples (e.g., PBMC ELISpot, fluorospot). Xiang et al [29] report experiments performed in a mouse model of T-cell activation that allows examination of hepatosplenic cellular immune responses. Their work suggests there may be significant nuance in the relationship between AAV vector CpG content, innate immune signaling, and the subsequent induction of anti-capsid T-cell response. Using AAV vectors containing relatively reduced or relatively enriched content of CpGs, they report that CpGs are essential to drive proliferation of naïve capsid-specific CD8+ T-cells, confirming a previous report from Faust and colleagues. In contrast, T-cell recall responses and proliferation of anti-capsid memory CD8+ T-cells appeared to be independent of innate immune responses, was not augmented by, and could even by attenuated by high vector CpG content. The studies suggest the possibility that capsid-specific de novo activated CD8+ T-cells mediate rejection of human AAV-transduced hepatocytes rather than recalled memory CD8+ T-cells.

Aside from the vector genome, the AAV capsid can also directly interact with the innate immune system. For example, it is known that TLR2 recognizes the AAV capsid peptides [50]. In addition to the membrane-bound TLRs, cytosolic DNA and RNA sensors play a role in virus recognition. It has been suggested that rAAV transduction could lead to formation of double-stranded RNA intermediates in the cytosol, which could stimulate innate immune signaling through cytosolic RNA sensors [76]. Additionally, preclinical studies have shown that complement proteins can directly bind to AAV vectors and may be a potential mediator of AAV vector immunogenicity [77,78]. Additional evidence comes from clinical studies of gene therapy for Duchenne muscular dystrophy (DMD). In these studies, very high doses of AAV vectors administered systemically resulted in complement activation [79,80], for which patients were treated with the complement component C5 inhibitor eculizumab [81,82].

### 3.3. Adaptive Immunity

Adaptive immune responses to AAV vector exposure include both B- and T-cell activation, which direct both antibody-mediated and cellular immunity.

Preexisting NAbs to AAV can have an important impact on the efficacy of gene transfer [12,67]. Given that NAb titers as low as 1:5 can completely block transduction of liver cells [47,83], the presence of antibodies to AAV is a common exclusion criterion in most liver-directed gene transfer trials. Despite the importance of measuring anti-AAV NAb titers in clinical trials in a consistent and comparable manner across studies, lack of standardization remains an issue for the field, preventing comparison of clinical outcomes in some cases. To this end, the development of standardized protocols for the measurement of anti-AAV antibodies will be crucial; such protocols should take into account the fact that differences in vector serotype, manufacturing method, and vector doses used in the clinic can affect the readout of antibody assays and the actual cutoff for inclusion in AAV trials.

As discussed above, the prevalence of NAbs to AAV vary with age of the individual, which may be important in the choice of the AAV serotype for clinical use in different populations (neonatal, pediatric, or adult) [4,28,84]. NAbs to AAV persist over time [17,63] and can effectively neutralize the AAV vector in gene therapy. Thus, NAbs remain an impediment to more widespread use of AAV gene therapy, as well as restrict the ability to re-administer the vector, if needed [4,28]. Cross-reactivity is also of concern, with most AAV serotypes, including AAV1, AAV2, AAV5, AAV6, AAV8, and AAV9, exhibiting varying degrees of cross-recognition by antibodies [4,58,59,85].

Although the presence of anti-AAV antibodies is expected to limit the efficacy of gene transfer, hemophilia trials have shown no evidence of toxicities associated with the presence of preexisting humoral immunity to the vector [12]. An ongoing Phase 3 AAV gene transfer trial in patients with hemophilia B did not observe a clear relationship between the emergence of treatment-related adverse events and the presence of detectable NAbs at baseline [34]. Conversely, trials in which large vector doses have been administered (e.g., >1 × 10^14^ vg/kg in trials of gene transfer for DMD [86]) have documented activation of complement, which may be mediated by antibody binding to a pathogen.

Cell-mediated immunity in the form of both T- and B-cell responses can also be triggered by the innate immune system, possibly through type I IFNs [4,28]. In contrast to animal models [87,88,89], human trials of AAV gene transfer have reported cases of liver toxicity that were attributed to a T-cell-mediated immune response [28]. This adverse effect appears to result from a dose-dependent cellular immune response [47]. Lower doses of the vector are more likely to result in mild inflammation, which is typically manageable with corticosteroids. Similar to the case with NAbs, there is evidence for cross-reactivity in cytotoxic T-lymphocyte (CTL) responses across multiple AAV serotypes [4,16,71]. Interestingly, there appears to be no correlation between humoral responses and AAV-specific T-cell responses [16,71,72], suggesting that participants lacking NAbs to an AAV vector may still manifest T-cell reactivity to AAV.

In clinical trials for hemophilia, T-cell responses to AAV have been associated with asymptomatic, self-limited increases in liver enzymes and loss of transgene expression. These effects have been documented across several studies, although in some cases, lack of transgene expression was not associated with detection of activated capsid-responsive T-cells in peripheral blood; similarly, detection of T-cells reactive to AAV was not associated with any clinically relevant observation [5,45].

A clear correlation between T-cell responses to AAV and liver expression of a transgene has been the subject of much debate. Various confounding factors may be responsible for this absence of a conclusive correlation between T-cell-mediated immunity and the outcome of gene transfer:

- T-cell reactivity to AAV has, in most trials, been measured with an IFN-γ enzyme- linked immune absorbent spot (ELISpot) assay, which has a limited readout, being focused on a single cytokine, and does not distinguish between T-cell subsets. The use of multicytokine ELISpot-based assays or more complex immunophenotyping of T-cells may help address this limitation.
- In all hemophilia gene transfer trials, immune responses have been monitored using circulating PBMCs, which may not represent the subset of T-cells residing in tissues transduced by AAV vectors. Recent progress toward characterizing and phenotyping of CD8+ T-cells that home to tissues, which may present specific surface markers detectable by flow cytometry [90], may help address this limitation of current immune-monitoring technologies.
- Importantly, the use of immunomodulatory regimens in gene transfer, although essential to maintain long-term expression following gene transfer, may limit or confound the ability to correlate immunology readouts with outcomes.

Several questions about T-cell responses observed in AAV gene transfer are still unanswered. Implementation of immune-monitoring protocols that include detailed phenotyping of T-cells and correlation of biomarkers of transgene expression and tissue damage remain important priorities in AAV trials that should help to elucidate the impact of T-cell responses on the outcome of AAV gene transfer in humans.

## 4. Mitigation Strategies to Overcome Vector Immunogenicity

Various strategies have been explored to overcome AAV vector immunogenicity.

Corticosteroids have been used broadly, either reactively or prophylactically, to manage T-cell reactivity to AAV in hemophilia trials. Although some trials showed that a short course of oral corticosteroids blocked an apparent capsid-driven T-cell response [10,11,20], this approach has not always been successful [4], even when high-dose corticosteroids were used [5,21]. In some cases, successful modulation of AAV immunogenicity has required the combination of corticosteroids with T-cell-targeting immunosuppressive drugs [91]. Although the use of various immunosuppressive drugs in gene transfer with AAV vectors has been proposed, preclinical studies indicate the importance of testing the safety of new regimens, particularly for liver-directed gene transfer. For example, the use of a three-drug anti-T-cell regimen designed to block the immune response, including the anti-IL-2 receptor antibody daclizumab [49], resulted in the consistent formation of inhibitory antibodies to human FIX following hepatic artery administration of an AAV vector expressing human FIX in nonhuman primates (NHPs). This study highlighted the importance of regulatory T-cells (Tregs) as mediators of transgene immune tolerance following hepatic gene transfer [92] and showed how drugs interfering with Treg induction could trigger a detrimental anti-transgene immune response [49,93,94].

Based on the findings of early clinical trials of gene transfer for hemophilia [10,12,16,20], corticosteroids and T-cell-targeting drugs have been used in several trials. More recently, the combination of both B- and T-cell-targeting drugs has been explored in humans (NCT01451879) to modulate AAV immunogenicity and allow for vector redosing. In addition, evidence that the complement pathway can mediate immune toxicities following AAV vector administration, at least at high vector doses, provides additional potential targets for immunomodulatory regimens in gene transfer [79,80,81].

Capsid engineering has also been proposed as a strategy to evade preexisting humoral immunity to AAV [95]. In addition to pharmacologic immunomodulation, vector engineering has the potential to greatly help reduce the immune response to AAV vectors. Strategies under evaluation in preclinical models and clinical trials include maximizing vector potency to decrease the therapeutic dose and the risk of immune-mediated toxicity, as well as increasing target cell transduction efficiency [96,97], transgene expression levels [44], or transgene activity [11,43].

Some transgene expression cassettes have been designed to decrease recognition of the viral genome by TLR9 so as to avoid activation of innate immune system responses [98].

Early clinical trial data support the role of innate immunity in AAV immunogenicity [5,30,82]. Examples of these bioengineering strategies include reducing the CpG content of the expression cassette via codon optimization or the engineering into the vector of sequences known to interfere with TLR-target binding (e.g., the (TTAGGG)_4_-like sequence derived from telomeres). It should be noted that, although codon optimization is now a standard tool used in recombinant protein expression systems to increase transgene expression, the introduction of synonymous nucleotide mutations can result in increased expression, decreased expression, or minimal change, and the effect of any change must be examined empirically [99,100]. Recently, attention has also turned to concern regarding the potential of synonymous mutations to impact the fidelity of pre-mRNA splicing, mRNA structure and stability, the rate of translation of protein from mRNA; the impact of these effects on protein folding and tertiary structure may theoretically affect protein function or immunogenicity. In fact, a naturally occurring synonymous mutation in the *F9* gene (G17736A/Val107Val) has been described as the underlying defect in a family manifesting defective factor IX activity; in this case, the conserved amino acid sequence FIX protein was not observed to be associated with immune recognition (e.g., inhibitor antibody formation) [101,102].

Modification of the AAV inverted terminal repeats (ITRs) to generate self-complementary (sc) AAVs has been used to increase expression upon transduction and allow transgene expression without the need for single-stranded (ss) DNA synthesis [98,103]. In some experimental models, FIX encoded by scAAV vectors is expressed earlier and at a higher level than when it is encoded by conventional ssAAV vectors [98,103,104]. Drawbacks of using scAAVs include their limited genome packaging capacity (~2.5 kb), which makes them unsuitable for packaging *F8* cDNA, and the possible higher level of activation of innate immunity compared with ssAAV vectors [24], given that dsDNA is a putative ligand for TLR9, whereas ssDNA is not a known PAMP for TLR binding. In the clinic, scAAVs have been used successfully in some AAV trials for hemophilia B [9,10,20,98].

Other potential strategies to evade the immune system response include engineering the promoter to increase transcription and optimizing the transgene codons to increase ribonucleic acid (RNA) production and translation (i.e., to achieve equivalent or better transgene expression with exposure to lower vector doses) [98]. However, there exist potential downsides to codon optimization that may affect protein conformation and stability, as well as protein function [105,106].

The influence of manufacturing on immunogenicity is being explored, including the role of empty capsids, which may not only serve as decoys for NAbs but also contribute to the number of capsid epitopes being presented to major histocompatibility complex (MHC) class I molecules. The presence of contaminants, such as host cell DNA and plasmid DNA, is another factor that may influence the immunogenicity of AAV vectors [47].

The modulation of T-cell responses in AAV gene transfer could be considered relatively straightforward, although it is important to assess the potential interactions between immunosuppressive agents and AAV vectors in suitable animal models [49]. The issue of humoral responses to AAV has been harder to tackle. One possible approach to mitigate the presence of NAbs to AAV is to administer high doses of AAV capsid [107]. However, although this strategy may be effective in subjects with low to moderate NAb titers, high capsid doses may elicit anti-capsid cytotoxic T-lymphocyte (CTL) responses [108]. Pharmacologic blockade of anti-capsid antibody formation following AAV vector administration has been extensively studied [109,110,111], with some early results in humans [112] and an open clinical study testing the possibility of re-administering AAV vectors in late onset Pompe disease patients (ClinicalTrials.gov NCT02240407) is currently open for enrollment.

**Figure 5:**
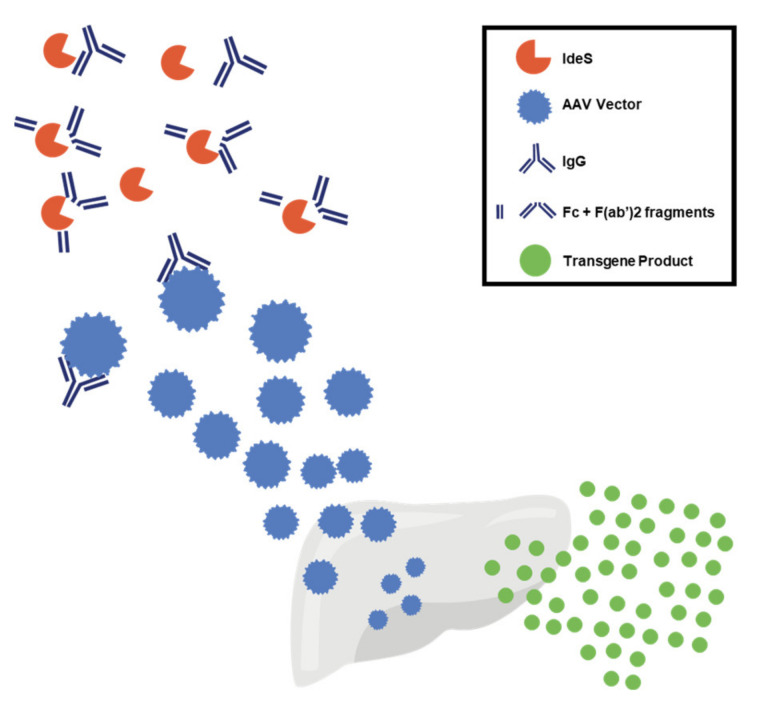
Mechanism of action of IdeS. IdeS is an IgG-degrading enzyme derived from *Streptococcus pyogenes* proposed as a strategy to overcome the limitation of neutralizing antibodies (NAbs) to AAV. IdeS is an endopeptidase that cleaves human IgG into F(ab′)2 and Fc fragments, thus reducing the neutralization activity of anti-AAV antibodies. AAV, adeno-associated virus; IdeS, imlifidase; IgG, immunoglobulin G; Fc, fragment crystallizable region; F(ab′)2, 2 antigen-binding (Fab) regions.

Removal of preexisting NAbs to AAV vectors has proven to be more challenging than prevention of antibody formation, mostly because of the limited number of drugs targeting antibody-producing plasma cells. Pharmacologic immunosuppression has had limited success in reducing anti-AAV NAbs in humans [113]; in contrast, the use of plasmapheresis has achieved promising results in preclinical animal models and humans [114,115,116,117,118,119]. Recently, the use of imlifidase (IdeS), an IgG-cleaving endopeptidase currently under investigation in solid organ transplantation [120,121], has been evaluated in AAV gene transfer (Figure 5). In preclinical studies, IdeS reduced anti-AAV antibody levels in vitro and in vivo and allowed for successful liver gene transfer in the settings of preexisting humoral immunity and vector re-administration [120,122]. An important caveat to these studies is the inclusion of models with only modest titers of NAbs. Additional analysis would be required in samples with high antibody titers, more reflective of potential participants in gene therapy redosing studies.

## 5. Inhibitors: Transgene-Related Immune Responses

Transgene-related immune responses are a potential important complication in the management of PWH, as the development of an inhibitor reduces the efficacy of clotting factor replacement and may necessitate the use of bypassing agents to control bleeding [123,124]. Inhibitors develop in 20 to 30% of people with severe hemophilia A who receive factor replacement therapy [42] and in 3 to 5% of those with severe hemophilia B who receive such therapy [125]. Preclinical animal models of gene therapy showed how host-specific factors, such as underlying inflammatory disease, immunity against self-protein, and immune system alterations, can affect the risk of developing inhibitors to the transgene product [4]. In mouse and dog models of hemophilia, the nature of host mutations in the *F8* and *F9* genes is a major determinant of the risk of inhibitor development in hemophilia gene transfer [51,126]. Vector-associated risks for inhibitors include the route of administration; specifically, intramuscular delivery may result in enhanced transgene immunogenicity, whereas hepatic administration reduces immunogenicity [127]. Another factor may be CpG-rich vector genomes [4]. To date, no incidences of inhibitor development have been reported in human gene transfer clinical trials for hemophilia. Of note, all participants enrolled in these trials were at low risk of inhibitor development, as they were previously exposed to clotting factors and had no active inhibitor at the time of AAV vector administration, making this a population with extremely low risk of mounting an immune response against the therapeutic transgene.

## 6. Immune Tolerance Induction

**Figure 6:**
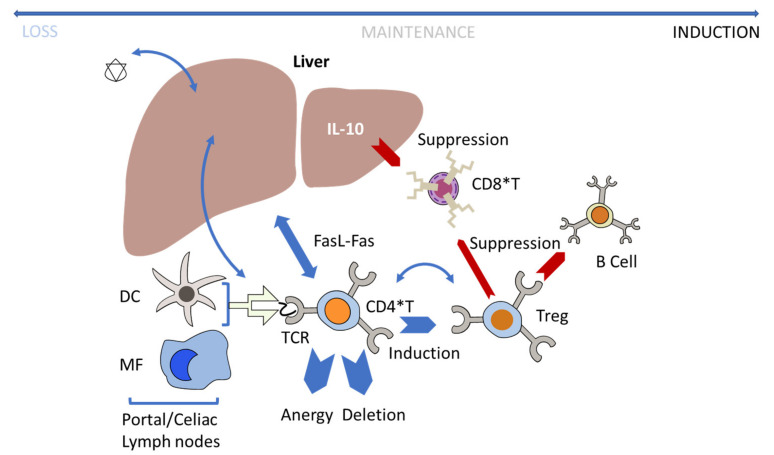
Antigen-specific transgene tolerance. Hepatic gene transfer with adeno-associated virus (AAV) vectors induces tolerance by multiple mechanisms, which include programmed cell death of CD4^+^ T-helper cells and the induction of FoxP3^+^ T_reg_. The initial presentation of antigens to the liver draining portal/celiac lymph nodes and the liver resident antigen-presenting cells (APCs) plays an important role in the induction of liver tolerance. DC, dendritic cell; Fas, Fas cell surface death receptor; FasL, Fas ligand; IL, interleukin; MF, macrophage; TCR, T-cell receptor; Treg, T regulatory cell.

Strategies to overcome or avoid transgene-related immune responses have been explored in preclinical trials. Liver-directed gene transfer appears to be less immunogenic than gene transfer to other tissues. Compared with other tissues, the liver microenvironment is unique, as exposure to foreign antigens through the gut does not necessarily result in an immune response. As such, immune responses driven by the liver are known to be limited, as demonstrated in transplant studies [127,128]. The tolerogenic effect of liver gene transfer is thought to stem from the difference in the antigen presentation context of the liver [129], which mediates various protolerogenic signals in gene transfer (Figure 6).

Both Kupffer cells (KCs) and dendritic cells (DCs) in the liver act as antigen-presenting cells (APCs) and have a less mature phenotype than peripheral APCs. As a result, KCs and DCs tend to be poor T-cell activators [4]. Moreover, KCs secrete IL-10, an anti-inflammatory cytokine, when stimulated by TLRs, and IL-10 suppresses CD8+ T-cell response. In addition, liver sinusoidal endothelial cells (LSECs) act as APCs and present antigens through MHC class II, which, in turn, induces Tregs [4,48].

### Regulatory T-Cells and Liver Tolerance

Several preclinical studies [49,130,131,132] have demonstrated that liver expression of a transgene can drive antigen-specific immunologic tolerance. In line with these preclinical studies, a case study documented the development of immunological tolerance to FVIII in a participant with severe hemophilia A and high-titer FVIII inhibitors following an orthotopic liver transplantation for hepatocellular carcinoma (HCC) in the setting of endogenous FVIII expression from the donor organ [133]. These results suggest that immune tolerance may be induced by the endogenous production of FVIII.

In the context of AAV gene transfer, it has been demonstrated that expression of human FIX in the liver of mice resulted in antigen-specific tolerance to the transgene product [130]. Similarly, several studies of AAV vectors in small and large animal models of genetic diseases showed that expression of various antigens in hepatocytes can promote robust antigen-specific immune tolerance [47,134,135]. Several laboratories investigating the mechanisms driving liver tolerance have pointed to Tregs as mediators of tolerance to liver-targeted transgenes [94,130,132,136,137,138]. Importantly, disruption of Treg homeostasis around the time of vector administration has been shown to lead to an immune response against the transgene [49,93,94]. Conversely, administration of rapamycin, a drug known to favor Treg expansion [139], enhanced the efficiency of tolerance induction in the context of established immunity [74,138].

Based on early research on the induction of immune tolerance via AAV gene transfer to the liver, several preclinical studies demonstrated that it is possible to use liver gene transfer to eradicate FVIII and FIX inhibitory antibodies. Eradication of these inhibitory antibodies has been achieved in both small [131] and large [134,140] preclinical models of hemophilia; the mechanism underlying this effect is thought to be steady-state hepatic expression of clotting factors via AAV vectors in previously immunized animals. Clinical translation of these results is planned, although there are no results so far (NCT03734588, NCT04684940).

## 7. AAV Vector Integration into the Host Genome

In addition to immunogenicity, a potential concern associated with gene-based therapies is the induction of genomic alterations leading to toxicities. Integration of wt.- AAV into the host cell genome may play a part in the ability of the virus to establish natural latency following infection; in addition, wt.-AAV expresses the protein Rep, which has DNA integrase activity [141,142]. Integration of wt.-AAV genomes has been found in humans [143,144], including the observed association of integrated AAV sequences in human HCC [143,144,145]. Recombinant AAV vectors do not contain any viral genes, do not encode the AAV Rep protein, and cannot efficiently integrate into the host DNA [47,72]. Nevertheless, AAV vectors have been shown to integrate with low efficiency into the host genome in animal models [146,147].

A study in a mouse model of mucopolysaccharidosis type VII (MPS VII), infused with recombinant AAV in the neonatal period and followed into adulthood, provided initial evidence that AAV genome integration can lead to HCC [148]. A subsequent study in which the factors driving AAV vector-associated risk of HCC formation (e.g., neonatal administration, high-vg/kg dose, strong viral promoter) were explored had similar findings [147]. In this study, a higher incidence of HCC was observed at higher vector doses, with constitutive promoters or promoters with high transactivating activity (particularly when compared to the lower risk posed by tissue-specific promoters), and in infused mice neonates. The mechanism postulated for the induction of HCC in mice via AAV vector integration was the dysregulation of microRNA-341 (Mir341) proximal to the Rian locus [147,148]. Indeed, the Mir341 locus appears to be a hotspot for AAV integration in the mouse genome. However, this locus has no ortholog in humans [149]. An 18-month follow-up study of adult mice given a FIX-expressing transgene driven by a liver-specific promoter found no evidence of insertional mutagenesis or cancer [150]. Similarly, no cases of HCC have been documented in dogs with hemophilia B treated with FIX gene therapy followed for 8 years [151] or in NHPs followed for 5 years [152]. Long-term follow-up of dogs with hemophilia A after high-dose gene transfer showed an absence of liver pathology but presence of nonmalignant clonal expansion of AAV-transduced hepatocytes resulting from the integration of the vector genome and subsequent homeostatic hepatocyte division [146,153]. Additional examination of integration events identified in this canine study demonstrated the vector genome was greatly truncated in the recovered sequences, in particular the *F8* transgene and the ITRs, i.e., no intact complete vector genome integration events were recovered [146]. Integration events were distributed throughout the dog chromosomes, with integration favoring sites associated with active transcription. Interestingly, the canine genome contains an ortholog of the *Rian* gene region; however, no integration events were observed in this gene region.

Several liver gene transfer studies with AAV vectors showed a favorable safety profile, with no reported treatment-related carcinogenic events [10,17,150,152]. In a recent hemophilia B trial, a participant given an AAV vector expressing FIX Padua was diagnosed with HCC 1 year following treatment; the study subject was asymptomatic, and the lesion was discovered on imaging performed as routine safety follow-up. Detailed investigations of the individual’s tumor and adjacent non-involved liver were performed including determination of the frequency and genomic location of AAV vector integration events; examination for clonal expansion of any vector sequences; whole genome sequencing of tumor to determine the presence of mutations characteristic of hepatocellular carcinoma independent of vector integration [154]. Consistent with findings from preclinical studies of rAAV integration, vector integration events in tumor were observed at a low frequency (estimated at 0.027% of cells in the tumor sample) and occurred randomly across the genome and without clonal expansion or dominant integration events. Whole genome sequencing provided additional convincing evidence that the development of HCC was not due to AAV vector integration but rather the result of the history of hepatitis virus B and C infection and nonalcoholic fatty liver disease (NAFLD) in this subject.

Overall, the risk of developing HCC following gene transfer with AAV vectors appears to be low. The extent to which underlying chronic hepatic inflammation (as was present in the clinical trial participant) may increase the risk of HCC following systemic AAV delivery [155] will require careful examination in human clinical trials, so that rare events are maximally informative. Vector design optimization (e.g., the use of tissue-specific promoters, minimizing total vg/kg exposure), long-term follow-up of trial participants (using liver imaging, with a low threshold for tissue examination) and capture of long-term outcomes in national and global gene therapy registries will be important steps to understand and improve the safety profile of AAV gene transfer.

## 8. Conclusions

Gene transfer is likely to play an increasingly important role in the treatment of genetic diseases such as hemophilia. Important challenges remain to be overcome, such as finding solutions to immune-related problems associated with viral vectors to provide safe, predictable, effective, and durable outcomes for PWH. Strategies to manage or better mitigate the immune response may include administration of transient immunomodulation and the use of agents such as IdeS. In addition, vector engineering and improvements in manufacturing may afford the opportunity to use lower vector doses, thereby decreasing immune-mediated toxicities. Lastly, long-term follow-up of people treated with AAV vectors [156] will add to our understanding of the durability of transgene expression in humans and the potential long-term risks associated with this therapy, including genotoxicity. Despite the challenges that remain to be overcome, the potential of gene transfer to improve therapeutic outcomes is significant. Novel frontiers, such as tolerance induction, show promise for the development of curative treatments for hemophilia.

## Figures and Tables

**Table 1:** Current FVIII AAV liver-directed gene transfer products for hemophilia A in clinical development.

Name	Sponsor	Vector Serotype	Transgene	Manufacturing	Phase	FVIII Range, % of Normal	ClinicalTrials.gov Identifier
Valoctocogene roxaparvovec(BMN-270) [8]	Biomarin	AAV5	FVIII-SQ	Baculovirus/insect cells	3	4–100 ^a^	NCT03370913 NCT03392974
SPK-8011 [35]	Spark	LK03	FVIII-SQ	Plasmid/mammalian cells	1/2	5.2–19.8 ^b^	NCT03003533
SPK-8016 [36]	Spark	NA	FVIII-SQ	Plasmid/mammalian cells	1/2	6–22	NCT03734588
AAV2/8-HLP-FVIII-V3 [37]	UCL	AAV8	FVIII-V3	Plasmid/mammalian cells	1/2	6–69	NCT03001830
SB-525 [38]	Sangamo/Pfizer	AAV6	FVIII-SQ	Baculovirus/insect cells	1/2	56.5–80.1	NCT03061201
TAK754 (BAX888) [39]	Takeda (Shire)	AAV8	FVIII-SQ	Plasmid/mammalian cells	1/2	NA	NCT03370172
BAY 2,599,023 (DTX201) [40]	Bayer	AAVhu37	FVIII-SQ	Plasmid/mammalian cells	1/2	5–17	NCT03588299

AAV, adeno-associated virus; FVIII, factor VIII; NA, not applicable; FVIII-SQ, B domain-deleted FVIII gene with SQ linker at site of deleted B domain sequence [41]; UCL, University College of London. ^a^ Three year follow up in Cohort 3. ^b^ > Two year follow up for the 5 × 10^11^ cohort and the 1 × 10^12^ cohort.

**Table 2:** Examples of AAV gene transfer clinical trials for hemophilia B.

Sponsor	Serotype/ Configuration	Number of CpG in ORF	Immune Suppression ^a^	CTL ^b^	Peak FIX	Duration
CHOP, Stanford Avigen [12,17]	AAV2-FIX/ss	19 (Wild type)	−	++	12% (*n* = 1)	<3 months
UCL, St. Jude [31]	AAV8-FIX/sc	0	+	+	2–11% (*n* = 10)	>7 years
Takeda (Shire) (BAX335) [5]	AAV8-FIX Padua/sc	99	++	++	4–58% (*n* = 8)	<3 months for 7/8 subjects >4 years for 1/8
CHOP	AAV8-FIX 19/ss	94 [22]	++	++ [22]	ND	ND
Pfizer (SPK-9001) [11]	AAVSPK-FIX Padua/ss	0	+	+	34% (*n* = 10)	>1 year
UniQure (AMT060) [32]	AAV5-FIX/sc	0	+	+	7% (*n* = 10)	>4 year
Dimension (DTX101) [21]	AAVrh10-FIX/ss	96	++	++	3–8% (*n* = 6)	<3 months
UniQure (AMT061) [34]	AAV5-FIX Padua/sc	0	−	+	36–51% (*n* = 3)	>2 years

AAV, adeno-associated virus; CHOP, Children’s Hospital of Philadelphia; CpG, cytosine triphosphate deoxynucleotide followed by guanine triphosphate deoxynucleotide with a phosphodiester link; CTL, cytotoxic T lymphocyte; ELISpot, enzyme-linked immunospot assay; FIX, factor IX; HEK, human embryonic kidney; IFN, interferon; ND, not determined; ORF, open reading frame; sc, self-complementary; ss, single strand; UCL, University College of London; ^a^ −, not used; +, minority of subjects; ++, majority of subjects; ^b^ capsid-specific CTLs by interferon gamma (IFN-γ) ELISpot: +, minority of subjects; ++, majority of subjects.

## Data Availability

Not applicable.

## References

[1] Nathwani A.C., Davidoff A.M., Tuddenham E.G.D. (2017). Advances in Gene Therapy for Hemophilia. Hum. Gene Ther..

[2] Doshi B.S., Arruda V.R. (2018). Gene therapy for hemophilia: What does the future hold?. Ther. Adv. Hematol..

[3] Srivastava A., Santagostino E., Dougall A., Kitchen S., Sutherland M., Pipe S.W., Carcao M., Mahlangu J., Ragni M.V., Windyga J. (2020). WFH Guidelines for the Management of Hemophilia, 3rd edition. Haemophilia.

[4] Verdera H.C., Kuranda K., Mingozzi F. (2020). AAV Vector Immunogenicity in Humans: A Long Journey to Successful Gene Transfer. Mol. Ther..

[5] Konkle B.A., Walsh C.E., Escobar M.A., Josephson N.C., Young G., von Drygalski A., McPhee S.W.J., Samulski R.J., Bilic I., de la Rosa M. (2021). BAX 335 hemophilia B gene therapy clinical trial results: Potential impact of CpG sequences on gene expression. Blood.

[6] Nathwani A.C., Davidoff A.M., Tuddenham E.G. (2017). Gene Therapy for Hemophilia. Hematol. Clin. N. Am..

[7] Peyvandi F., Garagiola I. (2019). Clinical advances in gene therapy updates on clinical trials of gene therapy in haemophilia. Haemophilia.

[8] Pasi K.J., Rangarajan S., Mitchell N., Lester W., Symington E., Madan B., Laffan M., Russell C.B., Li M., Pierce G.F. (2020). Multiyear Follow-up of AAV5-hFVIII-SQ Gene Therapy for Hemophilia A. N. Engl. J. Med..

[9] Miesbach W., Meijer K., Coppens M., Kampmann P., Klamroth R., Schutgens R., Tangelder M., Castaman G., Schwäble J., Bonig H. (2018). Gene therapy with adeno-associated virus vector 5–human factor IX in adults with hemophilia B. Blood.

[10] Nathwani A.C., Reiss U.M., Tuddenham E.G., Rosales C., Chowdary P., McIntosh J., Della Peruta M., Lheriteau E., Patel N., Raj D. (2014). Long-Term Safety and Efficacy of Factor IX Gene Therapy in Hemophilia B. N. Engl. J. Med..

[11] George L.A., Sullivan S.K., Giermasz A., Rasko J.E., Samelson-Jones B.J., Ducore J., Cuker A., Sullivan L.M., Majumdar S., Teitel J. (2017). Hemophilia B Gene Therapy with a High-Specific-Activity Factor IX Variant. N. Engl. J. Med..

[12] Manno C.S., Pierce G.F., Arruda V.R., Glader B., Ragni M., Rasko J.J., Ozelo M.C., Hoots K., Blatt P., Konkle B. (2006). Successful transduction of liver in hemophilia by AAV-Factor IX and limitations imposed by the host immune response. Nat. Med..

[13] Manno C.S., Chew A.J., Hutchison S., Larson P.J., Herzog R.W., Arruda V.R., Tai S.J., Ragni M.V., Thompson A., Ozelo M. (2003). AAV-mediated factor IX gene transfer to skeletal muscle in patients with severe hemophilia B. Blood.

[14] Simioni P., Tormene D., Tognin G., Gavasso S., Bulato C., Iacobelli N.P., Finn J.D., Spiezia L., Radu C., Arruda V.R. (2009). X-Linked Thrombophilia with a Mutant Factor IX (Factor IX Padua). N. Engl. J. Med..

[15] Monahan P.E., Sun J., Gui T., Hu G., Hannah W.B., Wichlan D.G., Wu Z., Grieger J.C., Li C., Suwanmanee T. (2015). Employing a Gain-of-Function Factor IX Variant R338L to Advance the Efficacy and Safety of Hemophilia B Human Gene Therapy: Preclinical Evaluation Supporting an Ongoing Adeno-Associated Virus Clinical Trial. Hum. Gene Ther..

[16] Mingozzi F., Maus M.V., Hui D.J., Sabatino D.E., Murphy S.L., Rasko J.E.J., Ragni M.V., Manno C.S., Sommer J., Jiang H. (2007). CD8+ T-cell responses to adeno-associated virus capsid in humans. Nat. Med..

[17] George L.A., Ragni M.V., Rasko J.E., Raffini L.J., Samelson-Jones B.J., Ozelo M., Hazbon M., Runowski A.R., Wellman J.A., Wachtel K. (2020). Long-Term Follow-Up of the First in Human Intravascular Delivery of AAV for Gene Transfer: AAV2-hFIX16 for Severe Hemophilia B. Mol. Ther..

[18] Gao G.-P., Alvira M.R., Wang L., Calcedo R., Johnston J., Wilson J.M. (2002). Novel adeno-associated viruses from rhesus monkeys as vectors for human gene therapy. Proc. Natl. Acad. Sci. USA.

[19] Nathwani A.C., Reiss U., Tuddenham E., Chowdary P., McIntosh J., Riddell A., Pie J., Mahlangu J.N., Recht M., Shen Y.-M. (2018). Adeno-Associated Mediated Gene Transfer for Hemophilia B:8 Year Follow up and Impact of Removing "Empty Viral Particles" on Safety and Efficacy of Gene Transfer. Blood.

[20] Nathwani A.C., Tuddenham E.G., Rangarajan S., Rosales C., McIntosh J., Linch D.C., Chowdary P., Riddell A., Pie A.J., Harrington C. (2011). Adenovirus-Associated Virus Vector–Mediated Gene Transfer in Hemophilia B. N. Engl. J. Med..

[21] Pipe S., Stine K., Rajasekhar A., Everington T., Poma A., Crombez E., Hay C.R. (2017). 101HEMB01 is a phase 1/2 open-label, single ascending dose-finding trial of DTX101 (AAVrh10FIX) in patients with moderate/severe hemophilia B that demonstrated meaningful but transient expression of human Factor IX (hFIX). Blood.

[22] High K.A., Anguela X. (2016). Modified Factor IX, and Compositions, Methods and Uses for Gene Transfer to Cells, Organs, and Tissues. U.S. Patent.

[23] Samelson-Jones B.J., Finn J.D., George L.A., Camire R.M., Arruda V.R. (2019). Hyperactivity of factor IX Padua (R338L) depends on factor VIIIa cofactor activity. JCI Insight.

[24] Martino A.T., Suzuki M., Markusic D.M., Zolotukhin I., Ryals R.C., Moghimi B., Ertl H.C.J., Muruve D.A., Lee B., Herzog R.W. (2011). The genome of self-complementary adeno-associated viral vectors increases Toll-like receptor 9–dependent innate immune responses in the liver. Blood.

[25] Rogers G.L., Shirley J.L., Zolotukhin I., Kumar S.R.P., Sherman A., Perrin G.Q., Hoffman B.E., Srivastava A., Basner-Tschakarjan E., Wallet M.A. (2017). Plasmacytoid and conventional dendritic cells cooperate in crosspriming AAV capsid-specific CD8+ T cells. Blood.

[26] Zhu J., Huang X., Yang Y. (2009). The TLR9-MyD88 pathway is critical for adaptive immune responses to adeno-associated virus gene therapy vectors in mice. J. Clin. Investig..

[27] Shirley J.L., Keeler G.D., Sherman A., Zolotukhin I., Markusic D.M., Hoffman B.E., Morel L.M., Wallet M.A., Terhorst C., Herzog R.W. (2020). Type I IFN Sensing by cDCs and CD4+ T Cell Help Are Both Requisite for Cross-Priming of AAV Capsid-Specific CD8+ T Cells. Mol. Ther..

[28] Kuranda K., Jean-Alphonse P., Leborgne C., Hardet R., Collaud F., Marmier S., Verdera H.C., Ronzitti G., Veron P., Mingozzi F. (2018). Exposure to wild-type AAV drives distinct capsid immunity profiles in humans. J. Clin. Investig..

[29] Xiang Z., Kurupati R.K., Li Y., Kuranda K., Zhou X., Mingozzi F., High K.A., Ertl H.C. (2020). The Effect of CpG Sequences on Capsid-Specific CD8+ T Cell Responses to AAV Vector Gene Transfer. Mol. Ther..

[30] Wright J.F. (2020). Quantification of CpG Motifs in rAAV Genomes: Avoiding the Toll. Mol. Ther..

[31] Nathwani A.C. (2019). Gene therapy for hemophilia. Hematology.

[32] Leebeek F.W., Meijer K., Coppens M., Kampmann P., Klamroth R., Schutgens M.R., Castaman G., Seifried E., Schwaeble J., Bönig H. (2020). AMT-060 Gene Therapy in Adults with Severe or Moderate-Severe Hemophilia B Confirm Stable FIX Expression and Durable Reductions in Bleeding and Factor IX Consumption for up to 5 Years. Blood.

[33] Von Drygalski A., Giermasz A., Castaman G., Key N.S., Lattimore S., Leebeek F.W.G., Miesbach W., Recht M., Long A., Gut R. (2019). Etranacogene dezaparvovec (AMT-061 phase 2b): Normal/near normal FIX activity and bleed cessation in hemophilia B. Blood Adv..

[34] Pipe S.W., Recht M., Key N.S., Leebeek F.W., Castaman G., Lattimore S.U., Van Der Valk P., Peerlinck K., Coppens M., O’Connell N. (2020). First Data from the Phase 3 HOPE-B Gene Therapy Trial: Efficacy and Safety of Etranacogene Dezaparvovec (AAV5-Padua hFIX variant; AMT-061) in Adults with Severe or Moderate-Severe Hemophilia B Treated Irrespective of Pre-Existing Anti-Capsid Neutralizing Antibodies. Blood.

[35] George L.E.E., Ragni M., Sullivan S., Samelson-Jones B., Evans M., MacDougall A., Curran M., Tompkins S., Wachtel K., Takefman D. Phase I/II Trial of SPK-8011: Stable and Durable FVIII Expression for >2 Years with Significant ABR Improvements in Initial Dose Cohorts Following AAV-Mediated FVIII Gene Transfer for Hemophilia A [abstract]. Res. Pract. Thromb. Haemost. 2020, 4 (Suppl 1). https://abstracts.isth.org/abstract/phase-i-ii-trial-of-spk-8011-stable-and-durable-fviii-expression-for-2-years-with-significant-abr-improvements-in-initial-dose-cohorts-following-aav-mediated-fviii-gene-transfer-for-hemophilia-a/.

[36] Sullivan S.K. (2021). SPK-8016: Preliminary Results From a Phase 1/2 Clinical Trial of Gene Therapy For Hemophilia A. in European Association for Haemophilia and Allied Disorders (EAHAD). Virtual Haemoph..

[37] Nathwani A.C., Tuddenham E., Chowdary P., McIntosh J., Lee D., Rosales C., Phillips M., Pie J., Junfang Z., Meagher M.M. (2018). GO-8: Preliminary Results of a Phase I/II Dose Escalation Trial of Gene Therapy for Haemophilia a Using a Novel Human Factor VIII Variant. Blood.

[38] Leavitt A.D., Konkle B.A., Stine K., Visweshwar N., Harrington T.J., Giermasz A., Arkin S., Fang A., Plonski F., Smith L. (2020). Updated Follow-up of the Alta Study, a Phase 1/2 Study of Giroctocogene Fitelparvovec (SB-525) Gene Therapy in Adults with Severe Hemophilia a. Blood.

[39] Chapin J. (2021). Results from a Phase 1/2 Safety and Dose Escalation Study of TAK-754, an AAV8 Vector With a Codon-Optimized B-Domain-Deleted Factor VIII Transgene in Severe Hemophilia A. in European Association for Haemophilia and Allied Disorders (EAHAD). Virtual Haemoph..

[40] Pipe S.W., Ferrante F., Reis M., Wiegmann S., Lange C., Braun M., Michaels L.A. (2020). First-in-Human Gene Therapy Study of AAVhu37 Capsid Vector Technology in Severe Hemophilia A-BAY 2599023 has Broad Patient Eligibility and Stable and Sustained Long-Term Expression of FVIII. Blood.

[41] Lind P., Larsson K., Spira J., Sydow-Bäckman M., Almstedt A., Gray E., Sandberg H. (1995). Novel Forms of B-Domain-Deleted Recombinant Factor VIII Molecules. Construction and Biochemical Characterization. JBIC J. Biol. Inorg. Chem..

[42] Samelson-Jones B.J., Arruda V.R. (2020). Translational Potential of Immune Tolerance Induction by AAV Liver-Directed Factor VIII Gene Therapy for Hemophilia A. Front. Immunol..

[43] McIntosh J., Lenting P.J., Rosales C., Lee D., Rabbanian S., Raj D., Patel N., Tuddenham E.G.D., Christophe O.D., McVey J.H. (2013). Therapeutic levels of FVIII following a single peripheral vein administration of rAAV vector encoding a novel human factor VIII variant. Blood.

[44] Ward N.N., Buckley S.M.K.S., Waddington S.S., VandenDriessche T., Chuah M.K.L., Nathwani A.A., McIntosh J.J., Tuddenham E.G.D.E., Kinnon C.C., Thrasher A. (2011). Codon optimization of human factor VIII cDNAs leads to high-level expression. Blood.

[45] Long B.R., Veron P., Kuranda K., Hardet R., Mitchell N., Hayes G.M., Wong W.Y., Lau K., Li M., Hock M.B. (2021). Early Phase Clinical Immunogenicity of Valoctocogene Roxaparvovec, an AAV5-Mediated Gene Therapy for Hemophilia A. Mol. Ther..

[46] Rangarajan S., Walsh L., Lester W., Perry D., Madan B., Laffan M., Yu H., Vettermann C., Pierce G.F., Wong W.Y. (2017). AAV5–Factor VIII Gene Transfer in Severe Hemophilia A. N. Engl. J. Med..

[47] Colella P., Ronzitti G., Mingozzi F. (2018). Emerging Issues in AAV-Mediated In Vivo Gene Therapy. Mol. Ther. Methods Clin. Dev..

[48] Shirley J.L., de Jong Y.P., Terhorst C., Herzog R.W. (2020). Immune Responses to Viral Gene Therapy Vectors. Mol. Ther..

[49] Mingozzi F., Hasbrouck N.C., Basner-Tschakarjan E., Edmonson S.A., Hui D.J., Sabatino D.E., Zhou S., Wright J.F., Jiang H., Pierce G.F. (2007). Modulation of tolerance to the transgene product in a nonhuman primate model of AAV-mediated gene transfer to liver. Blood.

[50] Hösel M., Broxtermann M., Janicki H., Esser K., Arzberger S., Hartmann P., Gillen S., Kleeff J., Stabenow D., Odenthal M. (2011). Toll-like receptor 2-mediated innate immune response in human nonparenchymal liver cells toward adeno-associated viral vectors. Hepatology.

[51] Fields P.A., Arruda V.R., Armstrong E., Chu K., Mingozzi F., Hagstrom J., Herzog R.W., High K.A. (2001). Risk and Prevention of Anti-factor IX Formation in AAV-Mediated Gene Transfer in the Context of a Large Deletion of F9. Mol. Ther..

[52] National Hemophilia Foundation (2020). HIV/AIDS. https://www.hemophilia.org/Bleeding-Disorders/Blood-Safety/HIVAIDS.

[53] Riley L., Womack W. (2012). Hepatitis and Hemophilia.

[54] Mazepa M.A., Monahan P.E., Baker J.R., Riske B.K., Soucie J.M. (2016). Men with severe hemophilia in the United States: Birth cohort analysis of a large national database. Blood.

[55] Hösel M., Lucifora J., Michler T., Holz G., Gruffaz M., Stahnke S., Zoulim F., Durantel D., Heikenwalder M., Nierhoff D. (2014). Hepatitis B virus infection enhances susceptibility toward adeno-associated viral vector transduction in vitro and in vivo. Hepatology.

[56] Srivastava A. (2016). Adeno-Associated Virus: The Naturally Occurring Virus Versus the Recombinant Vector. Hum. Gene Ther..

[57] Boutin S., Monteilhet V., Veron P., Leborgne C., Benveniste O., Montus M.F., Masurier C. (2010). Prevalence of Serum IgG and Neutralizing Factors Against Adeno-Associated Virus (AAV) Types 1, 2, 5, 6, 8, and 9 in the Healthy Population: Implications for Gene Therapy Using AAV Vectors. Hum. Gene Ther..

[58] Kruzik A., Fetahagic D., Hartlieb B., Dorn S., Koppensteiner H., Horling F.M., Scheiflinger F., Reipert B.M., de la Rosa M. (2019). Prevalence of Anti-Adeno-Associated Virus Immune Responses in International Cohorts of Healthy Donors. Mol. Ther. Methods Clin. Dev..

[59] Li C., Narkbunnam N., Samulski R.J., Asokan A., Hu G., Jacobson L.J., Manco-Johnson M.J., Monahan P.E. (2011). Neutralizing antibodies against adeno-associated virus examined prospectively in pediatric patients with hemophilia. Gene Ther..

[60] Gao G., Vandenberghe L.H., Alvira M.R., Lu Y., Calcedo R., Zhou X., Wilson J.M. (2004). Clades of Adeno-Associated Viruses Are Widely Disseminated in Human Tissues. J. Virol..

[61] Fitzpatrick Z., Leborgne C., Barbon E., Masat E., Ronzitti G., van Wittenberghe L., Vignaud A., Collaud F., Charles S., Sola M.S. (2018). Influence of Pre-existing Anti-capsid Neutralizing and Binding Antibodies on AAV Vector Transduction. Mol. Ther. Methods Clin. Dev..

[62] Mimuro J., Mizukami H., Shima M., Matsushita T., Taki M., Muto S., Higasa S., Sakai M., Ohmori T., Madoiwa S. (2014). The prevalence of neutralizing antibodies against adeno-associated virus capsids is reduced in young Japanese individuals. J. Med. Virol..

[63] Leborgne C., Latournerie V., Boutin S., Desgue D., Quéré A., Pignot E., Collaud F., Charles S., Sola M.S., Masat E. (2019). Prevalence and long-term monitoring of humoral immunity against adeno-associated virus in Duchenne Muscular Dystrophy patients. Cell. Immunol..

[64] Calcedo R., Vandenberghe L.H., Gao G., Lin J., Wilson J.M. (2009). Worldwide Epidemiology of Neutralizing Antibodies to Adeno-Associated Viruses. J. Infect. Dis..

[65] Liu Q., Huang W., Zhang H., Wang Y., Zhao J., Song A., Xie H., Zhao C., Gao D. (2014). Neutralizing antibodies against AAV2, AAV5 and AAV8 in healthy and HIV-1-infected subjects in China: Implications for gene therapy using AAV vectors. Gene Ther..

[66] Greenberg B.H., Butler J.A., Felker G.M., Ponikowski P., Voors A.A., Pogoda J.M., Provost R., Guerrero J.L., Hajjar R.J., Zsebo K.M. (2016). Prevalence of AAV1 neutralizing antibodies and consequences for a clinical trial of gene transfer for advanced heart failure. Gene Ther..

[67] Jiang H., Couto L.B., Patarroyo-White S., Liu T., Nagy D., Vargas J.A., Zhou S., Scallan C.D., Sommer J., Vijay S. (2006). Effects of transient immunosuppression on adenoassociated, virus-mediated, liver-directed gene transfer in rhesus macaques and implications for human gene therapy. Blood.

[68] Falese L., Sandza K., Yates B., Triffault S., Gangar S., Long B., Tsuruda L., Carter B., Vettermann C., Zoog S.J. (2017). Strategy to detect pre-existing immunity to AAV gene therapy. Gene Ther..

[69] Meliani A., Leborgne C., Triffault S., Jeanson-Leh L., Veron P., Mingozzi F. (2015). Determination of Anti-Adeno-Associated Virus Vector Neutralizing Antibody Titer with an In Vitro Reporter System. Hum. Gene Ther. Methods.

[70] Vandamme C., Xicluna R., Hesnard L., Devaux M., Jaulin N., Guilbaud M., Le Duff J., Couzinié C., Moullier P., Saulquin X. (2020). Tetramer-Based Enrichment of Preexisting Anti-AAV8 CD8+ T Cells in Human Donors Allows the Detection of a TEMRA Subpopulation. Front. Immunol..

[71] Hui D.J., Edmonson S.C., Podsakoff G.M., Pien G.C., Ivanciu L., Camire R.M., Ertl H., Mingozzi F., High K.A., Basner-Tschakarjan E. (2015). AAV capsid CD8+ T-cell epitopes are highly conserved across AAV serotypes. Mol. Ther. Methods Clin. Dev..

[72] Veron P., Leborgne C., Monteilhet V., Boutin S., Martin S., Moullier P., Masurier C. (2012). Humoral and Cellular Capsid-Specific Immune Responses to Adeno-Associated Virus Type 1 in Randomized Healthy Donors. J. Immunol..

[73] Trinchieri G., Sher A. (2007). Cooperation of Toll-like receptor signals in innate immune defence. Nat. Rev. Immunol..

[74] Herzog R.W., Cooper M., Perrin G.Q., Biswas M., Martino A.T., Morel L., Terhorst C., Hoffman B.E. (2019). Regulatory T cells and TLR9 activation shape antibody formation to a secreted transgene product in AAV muscle gene transfer. Cell. Immunol..

[75] Rogers G.L., Suzuki M., Zolotukhin I., Markusic D.M., Morel L.M., Lee B., Ertl H.C., Herzog R.W. (2015). Unique Roles of TLR9- and MyD88-Dependent and -Independent Pathways in Adaptive Immune Responses to AAV-Mediated Gene Transfer. J. Innate Immun..

[76] Shao W., Earley L.F., Chai Z., Chen X., Sun J., He T., Deng M., Hirsch M.L., Ting J., Samulski R.J. (2018). Double-stranded RNA innate immune response activation from long-term adeno-associated virus vector transduction. JCI Insight.

[77] Zaiss A.K., Cotter M.J., White L.R., Clark S.A., Wong N.C.W., Holers V.M., Bartlett J.S., Muruve D.A. (2008). Complement Is an Essential Component of the Immune Response to Adeno-Associated Virus Vectors. J. Virol..

[78] Denard J., Marolleau B., Jenny C., Rao T.N., Fehling H.J., Voit T., Svinartchouk F. (2013). C-Reactive Protein (CRP) Is Essential for Efficient Systemic Transduction of Recombinant Adeno-Associated Virus Vector 1 (rAAV-1) and rAAV-6 in Mice. J. Virol..

[79] Solid Biosciences Provides SGT-001 Program Update. https://www.solidbio.com/about/media/press-releases/solid-biosciences-provides-sgt-001-program-update.

[80] Duan D. (2018). Systemic AAV Micro-dystrophin Gene Therapy for Duchenne Muscular Dystrophy. Mol. Ther..

[81] (2018). Solid Biosciences Announces FDA Removes Clinical Hold on SGT-001. https://investors.solidbio.com/news-releases/news-release-details/solid-biosciences-announces-fda-removes-clinical-hold-sgt-001.

[82] Muhuri M., Maeda Y., Ma H., Ram S., Fitzgerald K.A., Tai P.W., Gao G. (2021). Overcoming innate immune barriers that impede AAV gene therapy vectors. J. Clin. Investig..

[83] Wang L., Calcedo R., Bell P., Lin J., Grant R.L., Siegel D.L., Wilson J.M. (2011). Impact of Pre-Existing Immunity on Gene Transfer to Nonhuman Primate Liver with Adeno-Associated Virus 8 Vectors. Hum. Gene Ther..

[84] Calcedo R., Morizono H., Wang L., McCarter R., He J., Jones D., Batshaw M.L., Wilson J.M. (2011). Adeno-Associated Virus Antibody Profiles in Newborns, Children, and Adolescents. Clin. Vaccine Immunol..

[85] Tseng Y.-S., Gurda B.L., Chipman P., McKenna R., Afione S., Chiorini J.A., Muzyczka N., Olson N.H., Baker T.S., Kleinschmidt J. (2014). Adeno-Associated Virus Serotype 1 (AAV1)- and AAV5-Antibody Complex Structures Reveal Evolutionary Commonalities in Parvovirus Antigenic Reactivity. J. Virol..

[86] Solid Biosciences Investor Presentation. Proceedings of the 39th Annual J.P. Healthcare Conference.

[87] Li C., Hirsch M., Asokan A., Zeithaml B., Ma H., Kafri T., Samulski R.J. (2007). Adeno-Associated Virus Type 2 (AAV2) Capsid-Specific Cytotoxic T Lymphocytes Eliminate Only Vector-Transduced Cells Coexpressing the AAV2 Capsid In Vivo. J. Virol..

[88] Li H., Murphy S.L., Giles-Davis W., Edmonson S., Xiang Z., Li Y., Lasaro M.O., High K.A., Ertl H.C. (2007). Pre-existing AAV Capsid-specific CD8+ T Cells are Unable to Eliminate AAV-transduced Hepatocytes. Mol. Ther..

[89] Gao G., Wang Q., Dai Z., Calcedo R., Sun X., Li G., Wilson J.M. (2008). Adenovirus based vaccines generate cytotoxic t lymphocytes to epitopes of ns1 from dengue virus that are present in all major serotypes. Hum. Gene Ther..

[90] Buggert M., Vella L.A., Nguyen S., Wu V., Chen Z., Sekine T., Perez-Potti A., Maldini C.R., Manne S., Darko S. (2020). The Identity of Human Tissue-Emigrant CD8+ T Cells. Cell.

[91] Nathwani A.C. Data update from the B-AMAZE Phase 1/2 Study–Verbrinacogene setparvovec (FLT180a). Freeline Corporate Presentation. https://www.freeline.life/media/1349/presentation-freeline-company-update-14-dec-2020.pdf.

[92] Sherman A., Biswas M., Herzog R.W. (2017). Innovative Approaches for Immune Tolerance to Factor VIII in the Treatment of Hemophilia A. Front. Immunol..

[93] Cao O., Dobrzynski E., Wang L., Nayak S., Mingle B., Terhorst C., Herzog R.W. (2007). Induction and role of regulatory CD4+CD25+ T cells in tolerance to the transgene product following hepatic in vivo gene transfer. Blood.

[94] Samelson-Jones B.J., Finn J.D., Favaro P., Wright J.F., Arruda V.R. (2020). Timing of Intensive Immunosuppression Impacts Risk of Transgene Antibodies after AAV Gene Therapy in Nonhuman Primates. Mol. Ther. Methods Clin. Dev..

[95] Tse L.V., Klinc K.A., Madigan V.J., Rivera R.M.C., Wells L.F., Havlik L.P., Smith J.K., Agbandje-McKenna M., Asokan A. (2017). Structure-guided evolution of antigenically distinct adeno-associated virus variants for immune evasion. Proc. Natl. Acad. Sci. USA.

[96] Lisowski L., Dane A.P., Chu K., Zhang Y., Cunningham S.C., Wilson E.M., Nygaard S., Grompe M., Alexander I.E., Kay M.A. (2014). Selection and evaluation of clinically relevant AAV variants in a xenograft liver model. Nat. Cell Biol..

[97] Ogden P.J., Kelsic E.D., Sinai S., Church G.M. (2019). Comprehensive AAV capsid fitness landscape reveals a viral gene and enables machine-guided design. Science.

[98] Li Ca Samulski R.J. (2020). Engineering adeno-associated virus vectors for gene therapy. Nat. Rev. Genet..

[99] Kimchi-Sarfaty C., Schiller T., Hamasaki-Katagiri N., Khan M.A., Yanover C., Sauna Z.E. (2013). Building better drugs: Developing and regulating engineered therapeutic proteins. Trends Pharmacol. Sci..

[100] Bali V., Bebok Z. (2015). Decoding mechanisms by which silent codon changes influence protein biogenesis and function. Int. J. Biochem. Cell Biol..

[101] Simhadri V.L., Hamasaki-Katagiri N., Lin B., Hunt R., Jha S., Tseng S.C., Wu A., Bentley A.A., Zichel R., Lu Q. (2017). Single synonymous mutation in factor IX alters protein properties and underlies haemophilia B. J. Med. Genet..

[102] Knobe K.E., Sjrin E., Ljung R.C.R., Sjörin E. (2008). Why does the mutationG17736AVal107Val (silent) in theF9gene cause mild haemophilia B in five Swedish families?. Haemophilia.

[103] Mccarty D.M., Monahan P.E., Samulski R.J. (2001). Self-complementary recombinant adeno-associated virus (scAAV) vectors promote efficient transduction independently of DNA synthesis. Gene Ther..

[104] Wu Z., Sun J., Zhang T., Yin C., Yin F., Van Dyke T., Samulski R.J., Monahan P.E. (2008). Optimization of Self-complementary AAV Vectors for Liver-directed Expression Results in Sustained Correction of Hemophilia B at Low Vector Dose. Mol. Ther..

[105] Mauro V.P., Chappell S.A. (2014). A critical analysis of codon optimization in human therapeutics. Trends Mol. Med..

[106] Alexaki A., Hettiarachchi G.K., Athey J.C., Katneni U., Simhadri V., Hamasaki-Katagiri N., Nanavaty P., Lin B., Takeda K., Freedberg D. (2019). Effects of codon optimization on coagulation factor IX translation and structure: Implications for protein and gene therapies. Sci. Rep..

[107] Mingozzi F., Anguela X.M., Pavani G., Chen Y., Davidson R.J., Hui D.J., Yazicioglu M., Elkouby L., Hinderer C.J., Faella A. (2013). Overcoming Preexisting Humoral Immunity to AAV Using Capsid Decoys. Sci. Transl. Med..

[108] Mingozzi F., High K.A. (2013). Immune responses to AAV vectors: Overcoming barriers to successful gene therapy. Blood.

[109] Meliani A., Boisgerault F., Hardet R., Marmier S., Collaud F., Ronzitti G., Leborgne C., Verdera H.C., Sola M.S., Charles S. (2018). Antigen-selective modulation of AAV immunogenicity with tolerogenic rapamycin nanoparticles enables successful vector re-administration. Nat. Commun..

[110] Corti M., Cleaver B., Clément N., Conlon T.J., Faris K.J., Wang G., Benson J., Tarantal A.F., Fuller D., Herzog R.W. (2015). Evaluation of Readministration of a Recombinant Adeno-Associated Virus Vector Expressing Acid Alpha-Glucosidase in Pompe Disease: Preclinical to Clinical Planning. Hum. Gene Ther. Clin. Dev..

[111] Unzu C., Hervás-Stubbs S., Sampedro A., Mauleón I., Mancheño U., Alfaro C., De Salamanca R.E., Benito A., Beattie S.G., Petry H. (2012). Transient and intensive pharmacological immunosuppression fails to improve AAV-based liver gene transfer in non-human primates. J. Transl. Med..

[112] Corti M., Elder M., Falk D., Lawson L., Smith B., Nayak S., Conlon T., Clément N., Erger K., Lavassani E. (2014). B-cell depletion is protective against anti-AAV capsid immune response: A human subject case study. Mol. Ther. Methods Clin. Dev..

[113] Mingozzi F., Chen Y., Edmonson S., Zhou S., Thurlings R.M., Tak P.P., High K.A., Vervoordeldonk M.J. (2012). Prevalence and pharmacological modulation of humoral immunity to AAV vectors in gene transfer to synovial tissue. Gene Ther..

[114] Salas D., Kwikkers K.L., Zabaleta N., Bazo A., Petry H., Van Deventer S.J., Aseguinolaza G.G., Ferreira V. (2019). Immunoadsorption enables successful rAAV5-mediated repeated hepatic gene delivery in nonhuman primates. Blood Adv..

[115] Monteilhet V., Saheb S., Boutin S., Leborgne C., Veron P., Montus M.-F., Moullier P., Benveniste O., Masurier C. (2011). A 10 Patient Case Report on the Impact of Plasmapheresis Upon Neutralizing Factors Against Adeno-associated Virus (AAV) Types 1, 2, 6, and 8. Mol. Ther..

[116] Bertin B., Veron P., Leborgne C., Deschamps J.-Y., Moullec S., Fromes Y., Collaud F., Boutin S., Latournerie V., Van Wittenberghe L. (2020). Capsid-specific removal of circulating antibodies to adeno-associated virus vectors. Sci. Rep..

[117] Orlowski A., Katz M.G., Gubara S.M., Fargnoli A.S., Fish K.M., Weber T. (2020). Successful Transduction with AAV Vectors after Selective Depletion of Anti-AAV Antibodies by Immunoadsorption. Mol. Ther. Methods Clin. Dev..

[118] Hurlbut G.D., Ziegler R.J., Nietupski J.B., Foley J.W., Woodworth L.A., Meyers E., Bercury S.D., Pande N.N., Souza D.W., Bree M.P. (2010). Preexisting Immunity and Low Expression in Primates Highlight Translational Challenges for Liver-directed AAV8-mediated Gene Therapy. Mol. Ther..

[119] Chicoine L., Montgomery C., Bremer W., Shontz K., Griffin D., Heller K., Lewis S., Malik V., Grose W., Shilling C. (2014). Plasmapheresis Eliminates the Negative Impact of AAV Antibodies on Microdystrophin Gene Expression Following Vascular Delivery. Mol. Ther..

[120] Leborgne C., Barbon E., Alexander J.M., Hanby H., Delignat S., Cohen D.M., Collaud F., Muraleetharan S., Lupo D., Silverberg J. (2020). IgG-cleaving endopeptidase enables in vivo gene therapy in the presence of anti-AAV neutralizing antibodies. Nat. Med..

[121] Jordan S.C., Lorant T., Choi J., Kjellman C., Winstedt L., Bengtsson M., Zhang X., Eich T., Toyoda M., Eriksson B.-M. (2017). IgG Endopeptidase in Highly Sensitized Patients Undergoing Transplantation. N. Engl. J. Med..

[122] Elmore Z.C., Oh D.K., Simon K.E., Fanous M.M., Asokan A. (2020). Rescuing AAV gene transfer from neutralizing antibodies with an IgG-degrading enzyme. JCI Insight.

[123] Kempton C.L., White G.C. (2009). How we treat a hemophilia A patient with a factor VIII inhibitor. Blood.

[124] Witmer C., Young G. (2012). Factor VIII inhibitors in hemophilia A: Rationale and latest evidence. Ther. Adv. Hematol..

[125] Castaman G., Matino D. (2019). Hemophilia A and B: Molecular and clinical similarities and differences. Haematology.

[126] Oldenburg J., Pavlova A. (2006). Genetic risk factors for inhibitors to factors VIII and IX. Haemophilia.

[127] Tiegs G., Lohse A.W. (2010). Immune tolerance: What is unique about the liver. J. Autoimmunity.

[128] Calne R.Y., Sells R.A., Pena J.R., Davis D.R., Millard P.R., Herbertson B.M., Binns R.M., Davies D.A.L. (1969). Induction of Immunological Tolerance by Porcine Liver Allografts. Nat. Cell Biol..

[129] Grakoui A., Crispe I.N. (2016). Presentation of hepatocellular antigens. Cell. Mol. Immunol..

[130] Mingozzi F., Liu Y.-L., Dobrzynski E., Kaufhold A., Liu J.H., Wang Y., Arruda V.R., High K.A., Herzog R.W. (2003). Induction of immune tolerance to coagulation factor IX antigen by in vivo hepatic gene transfer. J. Clin. Investig..

[131] Markusic D.M., Hoffman B.E., Perrin G.Q., Nayak S., Wang X., Loduca P.A., High K.A., Herzog R.W. (2013). Effective gene therapy for haemophilic mice with pathogenic factor IX antibodies. EMBO Mol. Med..

[132] Dobrzynski E., Mingozzi F., Liu Y.-L., Bendo E., Cao O., Wang L., Herzog R.W. (2004). Induction of antigen-specific CD4+ T-cell anergy and deletion by in vivo viral gene transfer. Blood.

[133] Ashrani A.A., Reding M.T., Shet A., Osip J., Humar A., Lake J.R., Key N.S. (2004). Successful liver transplantation in a patient with severe haemophilia A and a high-titre factor VIII inhibitor. Haemophilia.

[134] Finn J.D., Ozelo M.C., Sabatino D.E., Franck H.W.G., Merricks E.P., Crudele J.M., Zhou S., Kazazian H.H., Lillicrap D., Nichols T.C. (2010). Eradication of neutralizing antibodies to factor VIII in canine hemophilia A after liver gene therapy. Blood.

[135] Poupiot J., Verdera H.C., Hardet R., Colella P., Collaud F., Bartolo L., Davoust J., Sanatine P., Mingozzi F., Richard I. (2019). Role of Regulatory T Cell and Effector T Cell Exhaustion in Liver-Mediated Transgene Tolerance in Muscle. Mol. Ther. Methods Clin. Dev..

[136] Le Guen V., Judor J.-P., Boeffard F., Gauttier V., Ferry N., Soulillou J.-P., Brouard S., Conchon S. (2017). Alloantigen gene transfer to hepatocytes promotes tolerance to pancreatic islet graft by inducing CD8 + regulatory T cells. J. Hepatol..

[137] Breous E., Somanathan S., Wilson J.M. (2010). BALB/c Mice Show Impaired Hepatic Tolerogenic Response Following AAV Gene Transfer to the Liver. Mol. Ther..

[138] Keeler G.D., Kumar S., Palaschak B., Silverberg E.L., Markusic D.M., Jones N.T., Hoffman B.E. (2018). Gene Therapy-Induced Antigen-Specific Tregs Inhibit Neuro-inflammation and Reverse Disease in a Mouse Model of Multiple Sclerosis. Mol. Ther..

[139] Battaglia M., Stabilini A., Migliavacca B., Horejs-Hoeck J., Kaupper T., Roncarolo M.-G. (2006). Rapamycin Promotes Expansion of Functional CD4+CD25+FOXP3+ Regulatory T Cells of Both Healthy Subjects and Type 1 Diabetic Patients. J. Immunol..

[140] Crudele J.M., Finn J.D., Siner J.I., Martin N.B., Niemeyer G.P., Zhou S., Mingozzi F., Lothrop J.C.D., Arruda V.R. (2015). AAV liver expression of FIX-Padua prevents and eradicates FIX inhibitor without increasing thrombogenicity in hemophilia B dogs and mice. Blood.

[141] Smith R.H. (2008). Adeno-associated virus integration: Virus versus vector. Gene Ther..

[142] McCarty D.M., Young S.M., Samulski R.J. (2004). Integration of adeno-associated virus (AAV) and recombinant AAV vectors. Annu. Rev. Genet..

[143] Nault J.-C., Datta S., Imbeaud S., Franconi A., Mallet M., Couchy G., Letouzé E., Pilati C., Verret B., Blanc J.-F. (2015). Recurrent AAV2-related insertional mutagenesis in human hepatocellular carcinomas. Nat. Genet..

[144] La Bella T., Imbeaud S., Peneau C., Mami I., Datta S., Bayard Q., Caruso S., Hirsch T.Z., Calderaro J., Morcrette G. (2020). Adeno-associated virus in the liver: Natural history and consequences in tumour development. Gut.

[145] Nault J.-C., Mami I., La Bella T., Datta S., Imbeaud S., Franconi A., Mallet M., Couchy G., Letouzé E., Pilati C. (2016). Wild-type AAV Insertions in Hepatocellular Carcinoma Do Not Inform Debate Over Genotoxicity Risk of Vectorized AAV. Mol. Ther..

[146] Nguyen G.N., Everett J.K., Kafle S., Roche A.M., Raymond H.E., Leiby J., Wood C., Assenmacher C.-A., Merricks E.P., Long C.T. (2021). A long-term study of AAV gene therapy in dogs with hemophilia A identifies clonal expansions of transduced liver cells. Nat. Biotechnol..

[147] Chandler R.J., LaFave M.C., Varshney G.K., Trivedi N.S., Carrillo-Carrasco N., Senac J.S., Wu W., Hoffmann V., Elkahloun A.G., Burgess S.M. (2015). Vector design influences hepatic genotoxicity after adeno-associated virus gene therapy. J. Clin. Investig..

[148] Donsante A., Miller D., Li Y., Vogler C., Brunt E.M., Russell D.W., Sands M.S. (2007). AAV Vector Integration Sites in Mouse Hepatocellular Carcinoma. Science.

[149] Chandler R.J., LaFave M.C., Varshney G.K., Burgess S.M., Venditti C.P. (2016). Genotoxicity in Mice Following AAV Gene Delivery: A Safety Concern for Human Gene Therapy?. Mol. Ther..

[150] Li H., Malani N., Hamilton S.R., Schlachterman A., Bussadori G., Edmonson S.E., Shah R., Arruda V.R., Mingozzi F., Wright J.F. (2011). Assessing the potential for AAV vector genotoxicity in a murine model. Blood.

[151] Niemeyer G.P., Herzog R.W., Mount J., Arruda V.R., Tillson D.M., Hathcock J., Van Ginkel F.W., High K.A., Lothrop C.D. (2009). Long-term correction of inhibitor-prone hemophilia B dogs treated with liver-directed AAV2-mediated factor IX gene therapy. Blood.

[152] Nathwani A.C., Rosales C., McIntosh J., Rastegarlari G., Nathwani D., Raj D., Nawathe S., Waddington S.N., Bronson R., Jackson S. (2011). Long-term Safety and Efficacy Following Systemic Administration of a Self-complementary AAV Vector Encoding Human FIX Pseudotyped With Serotype 5 and 8 Capsid Proteins. Mol. Ther..

[153] Venditti C.P. (2021). Safety questions for AAV gene therapy. Nat. Biotechnol..

[154] uniQure (2021). uniQure Announces FDA Removes Clinical Hold on Hemophilia B Gene Therapy Program. https://tools.eurolandir.com/tools/Pressreleases/GetPressRelease/?ID=3902770&lang=en-GB&companycode=nl-qure&v=.

[155] Dalwadi D.A., Torrens L., Abril-Fornaguera J., Pinyol R., Willoughby C., Posey J., Llovet J.M., Lanciault C., Russell D.W., Grompe M. (2021). Liver Injury Increases the Incidence of HCC following AAV Gene Therapy in Mice. Mol. Ther..

[156] Konkle B.A., Recht M., Hilger A., Marks P. (2021). The critical need for postmarketing surveillance in gene therapy for haemophilia. Haemophilia.

